# Automated Research Platform for Development of Triplet–Triplet Annihilation Photon Upconversion Systems

**DOI:** 10.1021/acscentsci.4c02059

**Published:** 2025-02-21

**Authors:** Paulius Baronas, Justas Lekavičius, Maciej Majdecki, Jacob Lynge Elholm, Karolis Kazlauskas, Przemysław Gaweł, Kasper Moth-Poulsen

**Affiliations:** † Department of Chemical Engineering, Universitat Politècnica de Catalunya, EEBE, Eduard Maristany 10-14, 08019 Barcelona, Spain; ‡ Institute of Photonics and Nanotechnology, Vilnius University, Saulėtekis av. 3, 10257 Vilnius, Lithuania; § Institute of Organic Chemistry, Polish Academy of Sciences, Kasprzaka 44/52, 01-224 Warsaw, Poland; ∥ Catalan Institution for Research & Advanced Studies, ICREA, Pg. Lluís Companys 23, 08010 Barcelona, Spain; ⊥ Department of Chemistry and Chemical Engineering, Chalmers University of Technology, SE-41296 Gothenburg, Sweden; # The Institute of Materials Science of Barcelona, ICMAB-CSIC, Bellaterra, 08193 Barcelona, Spain

## Abstract

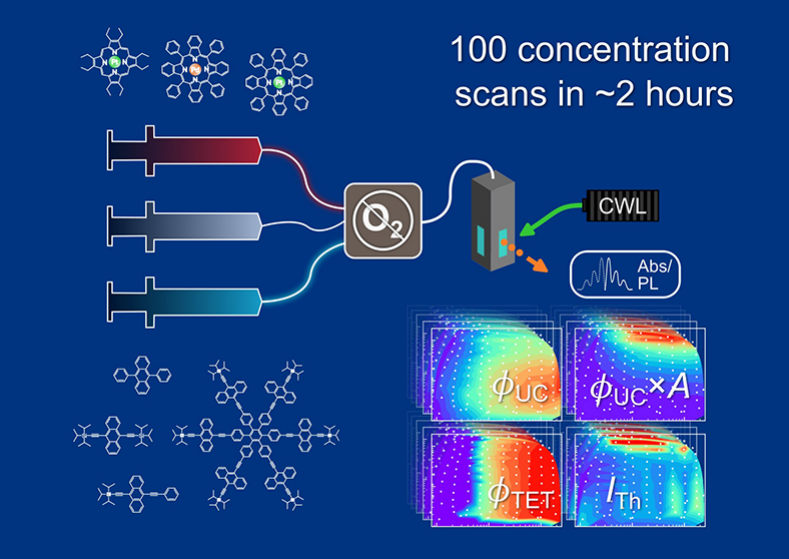

Triplet–triplet annihilation photon upconversion (TTA-UC) systems hold great promise for applications in energy, 3D printing, and photopharmacology. However, their optimization remains challenging due to the need for precise tuning of sensitizer and annihilator concentrations under oxygen-free conditions. This study presents an automated, high-throughput platform for the discovery and optimization of TTA-UC systems. Capable of performing 100 concentration scans in just two hours, the platform generates comprehensive concentration maps of critical parameters, including quantum yield, triplet energy transfer efficiency, and threshold intensity. Using this approach, we identify key loss mechanisms in both the established and novel TTA-UC systems. At high porphyrin-based sensitizer concentrations, upconversion quantum yield losses are attributed to sensitizer triplet self-quenching via aggregation and sensitizer triplet–triplet annihilation (sensitizer-TTA). Additionally, reverse triplet energy transfer (RTET) at elevated sensitizer levels increases the upconversion losses and excitation thresholds. Testing novel sensitizer–annihilator pairs confirms these loss mechanisms, highlighting opportunities for molecular design improvements. This automated platform offers a powerful tool for advancing TTA-UC research and other photochemical studies requiring low oxygen levels, intense laser excitation, and minimal material use.

## Abstract 2

A high-throughput concentration screening method built from commercial components for determining upconversion quantum yield and excitation threshold in sensitized triplet−triplet annihilation systems.

## Introduction

Triplet–triplet annihilation photon upconversion (TTA-UC) is a photophysical process of profound scientific interest as it facilitates the incoherent UC of low-energy photons at relatively low excitation densities.^1^ Over the past decades, significant advances in molecular design have maximized the so-called anti-Stokes shift, enabling near-infrared to visible and visible to ultraviolet light UC, increased UC efficiency above 30%, and a reduced excitation threshold closer to solar illumination intensity.^2,3^ These achievements attracted interest in many applications, including solar energy harvesting systems,^4^ photochemistry,^5^ life science applications,^6^ additive manufacturing,^7,8^ and excitonic logic.^9^ While many molecular design studies focus on tuning molecular parameters for optimal performance, practical applications often require operation in suboptimal conditions (i.e., high constituent concentrations, ambient oxygen, or low excitation densities), where loss management can play a major role. The high number of variables involved in TTA-UC analysis indicates significant potential for automated high-throughput experimentation.

The past decade has shown numerous examples of accelerated discovery of previously unexplored variable chemical spaces through automated experimentation.^10−12^ Due to the high number of variables and wide applicability, initial efforts have focused on automating chemical synthesis.^13−16^ However, more recent examples have integrated the characterization of target physicochemical properties into the automated discovery framework. Notable examples include automated screening platforms for organic lasers,^17^ organic solar cells,^18^ quantum dots,^19^ and perovskites.^20,21^ Automated characterization not only simplifies laborious tasks and substantially saves materials but also enables the discovery of new phenomena by generating large data sets effortlessly. In many cases it led to deeper insights into the materials’ characteristics compared to what would have been practically possible with traditional experimentation.

In this work, we developed an automated research platform for screening the multicomponent space of the TTA-UC systems. This platform performs concentration mapping of relevant parameters in various molecular sensitizer–annihilator combinations. Screening concentrations across up to 3 orders of magnitude in each component direction allowed us to identify optimal parameters for the highest UC efficiency of known TTA-UC systems and compare them to previously reported results. Such unprecedented mapping capability also enabled the visual identification of loss mechanisms in regions of suboptimal conditions and their correlation with specific molecular properties. The scope of this platform is additionally demonstrated through the identification of new excitonic relaxation pathways in four novel systems. The results obtained from the automated TTA-UC screening were validated independently using time-resolved techniques at significant concentrations. Ultimately, these findings enabled us to formulate guidelines for optimizing practical TTA-UC systems, providing valuable insights for future researchers.

## TTA-UC Parameters

**Figure 1:**
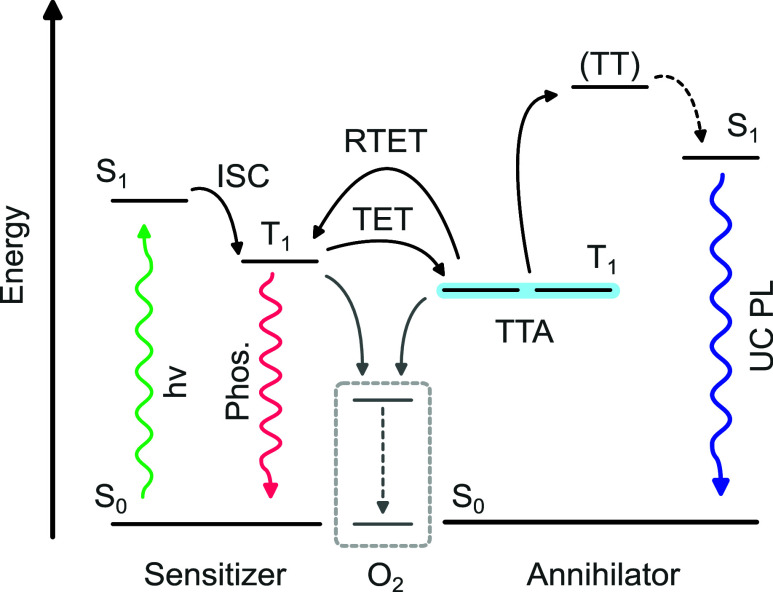
Photon energy upconversion diagram of a triplet–triplet annihilation system.

TTA-UC is a multiexciton process in which the energy of two photons is combined to produce one higher-energy photon. Initially, the low-energy photons (*h*ν) are absorbed by the photosensitizer (PS), where the energy is converted to a long-lived triplet excited state via intersystem crossing (ISC, Figure 1). Upon formation, the triplet excited state is transferred to the annihilator molecules through a Dexter-type triplet energy transfer (TET). The rates of TET and reverse triplet energy transfer (RTET) depend on the relative energy gaps between the triplet states of the sensitizer and the annihilator as well as their concentrations. This concentration dependence arises because collisions between sensitizers and annihilators in liquid solutions are limited by molecular diffusion. Eventually, after the subsequent collision of two excited annihilator molecules at their triplet state, TTA occurs, generating an excited annihilator singlet state, which emits a photon that is “anti-Stokes” shifted relative to the excitation light. An important point is that ambient oxygen quenches the triplet excited state inhibiting TTA-UC. This and other competing loss pathways can substantially reduce TTA-UC efficiency, which often exhibits complex, concentration-dependent behavior.^22,23^

The effective UC emission quantum yield (*ϕ*_UC_) is a combination of multiple processes and can be calculated using the following equation:
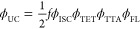
1where *ϕ*_ISC_, *ϕ*_TET_, *ϕ*_TTA_, and *ϕ*_FL_ are the quantum yields of ISC, TET, TTA, and annihilator fluorescence, respectively, and 1/2 represents that two annihilator triplet excitations are necessary to generate one singlet excitation (i.e., 50% maximum quantum yield).^24^ Intramolecular parameters of sensitizer and annihilator (e.g., *ϕ*_ISC_ and *ϕ*_FL_) are determined independently for each compound. A wide selection of sensitizers with high ISC rates and annihilators with high radiative rates have been explored for TTA-UC.^4,25,26^ On the other hand, the optimization of intermolecular parameters (e.g., *ϕ*_TET_ and *ϕ*_TTA_) requires alignment of triplet energy levels between sensitizer and annihilator, adjustment of components’ concentrations, and high excitation photon density at the same time. *ϕ*_TET_ is estimated as a sensitizer’s phosphorescence quenching efficiency from a sensitizer’s phosphorescence quantum yield (*ϕ*_Phos_) or its lifetime (τ_Phos_) with and without the presence of annihilator in the UC system (TET increases with annihilator concentration):^27^
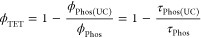
2

Even if all the quantum yields in eq 1 are optimized to approach unity, the total *ϕ*_UC_ remains limited by the spin-statistical factor *f*, which is an inherent characteristic of an annihilator molecule.^4,27^ It is defined as the probability that two low-energy triplet states will combine to form a single high-energy triplet pair state (TT) with an overall singlet character.^28^ In practice, values as high as 77% have been reported for TIPS-anthracene.^29^

The nonlinear behavior of bimolecular TTA indicates strong dependence of *ϕ*_TTA_ (and *ϕ*_UC_) on excitation density. *ϕ*_UC_ approaches its maximum value at excitation densities where *ϕ*_TTA_ is unity.^30^ Another important TTA-UC parameter derived from excitation density dependence is the excitation intensity threshold (*I*_th_(50%)), which corresponds to the point where *ϕ*_UC_ reaches half of its maximum value.^30^

## Results and Discussion

### Design of Automated TTA-UC Platform

**Figure 2:**
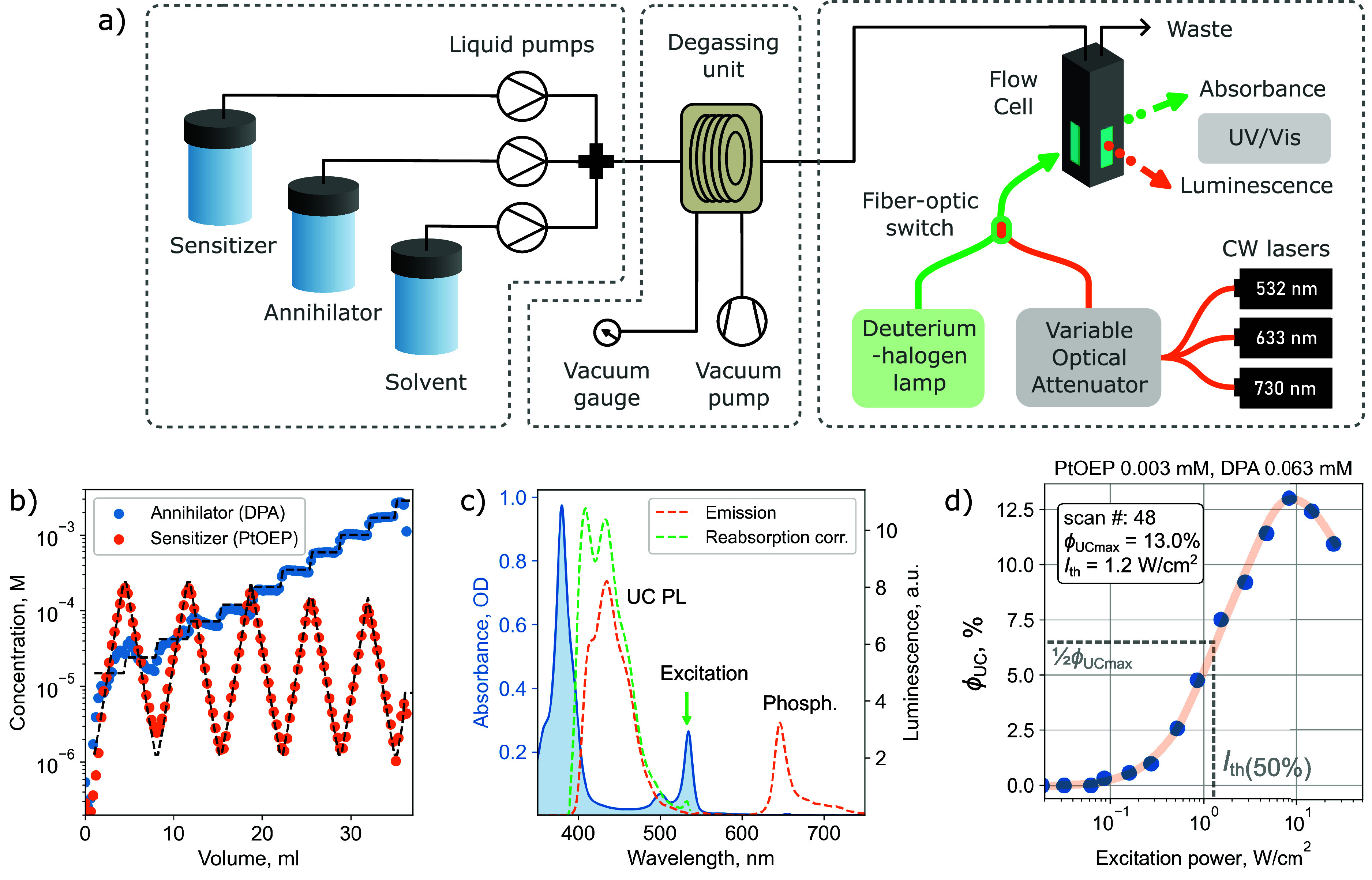
(a) Schematic representation of the automated TTA-UC screening platform. Liquid sampling, degassing, and sample spectroscopic characterization stages are separated by dashed frames. Detailed explanation of the automated platform is provided in the Supporting Information. (b) Concentrations estimated from absorbance measurements (full circles) and preset concentrations (dashed lines) of the sensitizer and annihilator. Each symbol represents a separate measurement, and volume corresponds to total solvent consumed. (c) Recorded absorbance and photoluminescence spectra. Upconverted PL was corrected for reabsorption due to considerable overlap with absorption. (d) Excitation power dependence of upconversion quantum yield *ϕ*_UC_ measured at specified sensitizer–annihilator concentrations. Excitation threshold density *I*_th_(50%) in W/cm^2^ is identified at half of the maximum *ϕ*_UC_.

Given the complexity of the multicomponent TTA-UC systems described above, comprehensively analyzing new systems poses significant challenges, highlighting the need for practical solutions to facilitate their discovery. To minimize the time required for full characterization of TTA-UC sensitizer–annihilator pairs at different conditions, we developed a platform to enable automated control of component concentrations, oxygen level, and photon density (see Section S2 for more details). Our automated TTA-UC screening platform is based on three stages of continuous liquid flow operation (Figure 2a): (1) precise liquid sampling of stock solutions, (2) removal of oxygen in the degassing unit, and (3) spectroscopic characterization of TTA-UC parameters. Additional benefit of continuous flow over batch testing is the possibility to measure across concentration gradients while maintaining constant oxygen concentration throughout all measurements.

In the first stage, the previously prepared stock solutions of the sensitizer and annihilator are pumped together with the solvent to produce accurate blends. Screening of sensitizer–annihilator concentrations is performed in constantly increasing annihilator concentration, while sensitizer concentration is changed in a “zig-zag” pattern (see Figure 2b) to avoid large concentration gradients between each segment. After sensitizer–annihilator blending, oxygen is removed from samples in continuous liquid flow using a high efficiency degassing method developed in our previous work.^31^ Residual oxygen concentration in measured solutions was determined to be down to 5 μM (see Section S2 for details on the oxygen concentration estimation).

Spectroscopic characterization of the degassed solution is performed in a three-way flow cell for simultaneous recording of photoluminescence and absorbance spectra. The absorbance measurements were used to validate that concentrations were correctly set in the blending stage. Excitation density was controlled with a variable optical attenuator (VOA) coupled to one of the three continuous wave (CW) lasers of 532, 633, and 730 nm emission wavelengths which match the absorption spectra of the most popular TTA-UC sensitizers.

The estimation of *ϕ*_UC_ was performed using the relative emission quantum yield method by comparing emission intensity with a reference standard (see Section S3 for more details). As noted by Zhou et al., the observed *ϕ*_UC_ can be different from the intrinsic one due to light outcoupling.^24^ In our setup, optical outcoupling is influenced by emission reabsorption in the path from the point where the laser excitation light is absorbed and UC occurs to the point where UC emission exits the flow cell. The simultaneous acquisition of absorption and emission spectra enforced reabsorption correction of UC emission spectra as presented in Figure 2c. Additionally, a correction for optical incoupling was performed to address the shorter light path at high sensitizer concentrations. This adjustment was necessary to account for the low emission collection efficiency in strongly absorbing samples (Figure S3).

### Concentration Mapping of PtOEP-DPA

**Figure 3:**
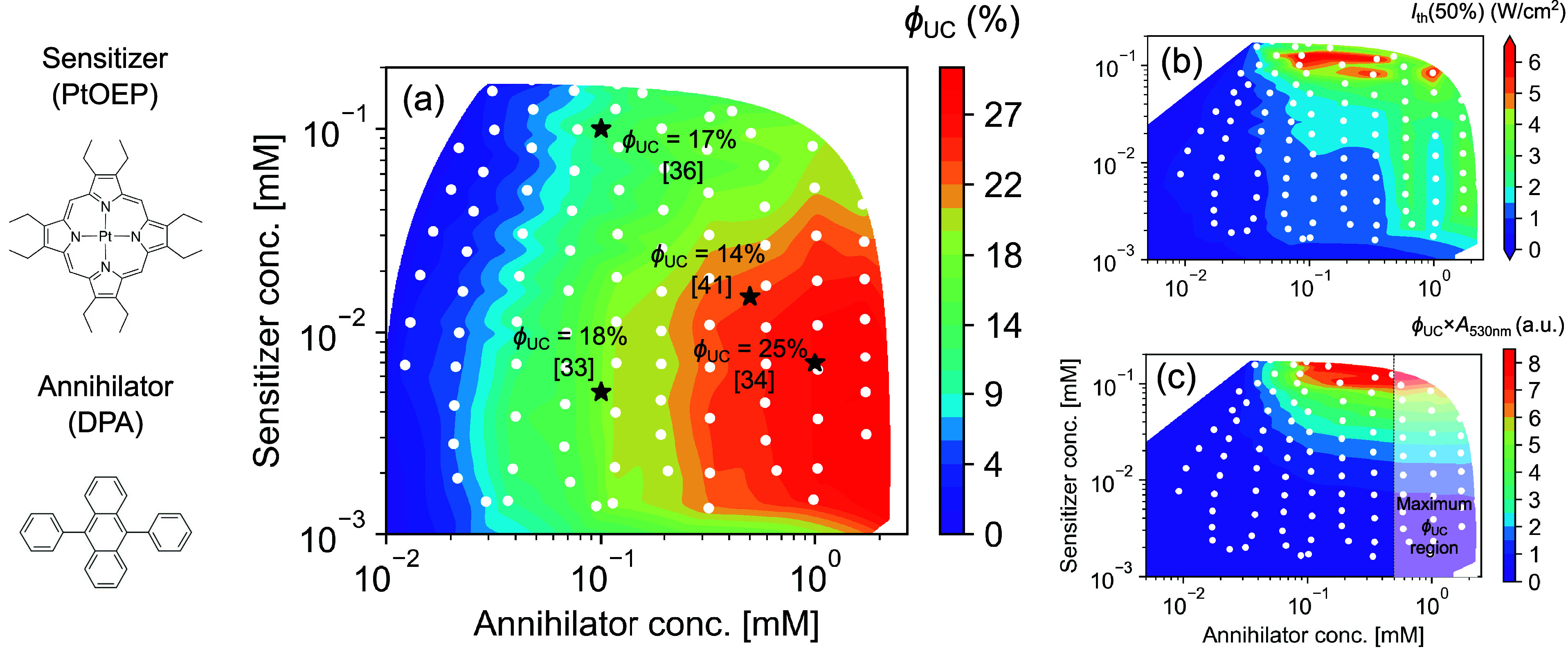
Concentration mapping of main TTA-UC parameters for the PtOEP-DPA system in toluene. (a) Map of upconversion quantum yield *ϕ*_UC_ (50% maximum) as a function of sensitizer–annihilator concentration. Literature *ϕ*_UC_ values of the PtOEP-DPA system measured at specific concentrations are indicated by black stars with the corresponding reference in parentheses. (b) Map of excitation intensity threshold (*I*_th_(50%)) in power density units W/cm^2^. (c) Map of emission output per incident photon at a peak absorbance wavelength. Dashed line indicates 0.5 mM annihilator concentration above which a negligible increase in emission output is observed. White dots indicate 121 measured concentration combinations.

For initial testing of the automated TTA-UC screening platform, we selected a well-studied sensitizer–annihilator pair of platinum octaethylporphyrin (PtOEP) and 9,10-diphenylanthracene (DPA).^32,33^ The concentration mapping of sensitizer and annihilator relevant parameters is presented in Figure 3. Exact concentrations of both components (as presented in Figure 2b) were determined using the Beer–Lambert law from measured absorption and corresponding molar extinction coefficients. Simultaneously, measured emission spectra were corrected for reabsorption and integrated in the 390–540 nm region (Figure 2c) to obtain UC emission intensity. The UC emission intensity per absorbed photon was used to determine *ϕ*_UC_ using the relative quantum yield method, with Rhodamine B as the fluorescent standard (Section S3). We also estimated UC emission intensity per incident photon at maximum absorbance (*ϕ*_UC_ × *A*_max_), a parameter that corresponds to maximum brightness of the sample. Finally, *I*_th_(50%) values were determined as the laser excitation power density at 1/2 *ϕ*_UC_^max^ (Figure 2d).

The concentration mapping was constructed from 121 data points, carried out during a period of time of only 165 min. Moreover, due to the miniaturized nature of the flow cell, containing only a 50 μL volume, all experiments could be carried out with less than 5 mL of sensitizer and annihilator stock solutions and 30 mL of solvent. Compared to manual cuvette-based experiments, which typically would consume between 0.5 and 1 mL of solvent per sample, automated screening could lead to significant material savings.

Obtained *ϕ*_UC_ values as a function of sensitizer–annihilator molar concentration are presented on smoothed contour surface shown in Figure 3a. The *ϕ*_UC_ reached 27% at sensitizer and annihilator (PtOEP:DPA) concentrations of 10 μM and 2 mM, respectively. Olesund et al. reported a *ϕ*_UC_ of 25% at 7 μM PtOEP and 1 mM DPA concentrations,^34^ which is in excellent agreement with values obtained using the automated TTA-UC screening platform. Yanai reported that at even higher PtOEP (100 μM) and DPA (10 mM) concentrations *ϕ*_UC_ is approaching 18% (reabsorption corrected),^35^ which indicates that peak *ϕ*_UC_ are already reached at 2 mM annihilator concentration as measured with this automated system.

It must be noted that *ϕ*_UC_ values reach a maximum at high excitation densities (1–10 W/cm^2^), which is required for the TTA rate to overcome the oxygen quenching rate. Importantly, significant variation of reported *ϕ*_UC_ values in the literature could also be caused by different oxygen removal methods, degradation, or different reabsorption correction protocols used. However, the automated TTA-UC screening platform enabled identification of suboptimal values. We thoroughly reviewed the literature to find reported *ϕ*_UC_ values measured at nonoptimal sensitizer–annihilator (PtOEP-DPA) molar concentration and plotted them on Figure 3a. From the optimization point of view, there are several areas of the concentration map to be discussed below.

At suboptimal annihilator concentrations, we found good agreement with Khnayzer et al., who reported a *ϕ*_UC_ = 18% measured at 5 μM PtOEP and 0.1 mM DPA.^33^ The decline of *ϕ*_TET_ at annihilator concentrations below 0.1 mM was confirmed by concentration mapping of the sensitizer phosphorescence quantum yield (*ϕ*_Phos_) (Figure S4). At low annihilator concentration, a drop in *ϕ*_UC_ indicates inefficient TET or quenching by impurities (e.g., oxygen).

At higher sensitizer concentration (above 0.1 mM), it acts itself as quencher leading to a *ϕ*_UC_ drop of approximately 30–40% (Figure 3a). Monguzzi et al. reported that at 100 μM PtOEP concentration *ϕ*_UC_ values were approaching 17% (0.1 mM DPA) at high excitation density conditions.^36^ At such high sensitizer concentrations, several previously reported loss mechanisms may come into play.

- Förster resonance energy transfer (FRET) from excited annihilator singlet state to sensitizer singlet state reduces the *ϕ*_PL_ of the annihilator and overall *ϕ*_UC_ of the TTA-UC system (eq 1). In this case, FRET-induced quenching could be caused by nonzero spectral overlap between DPA emission and PtOEP absorbance (see Figure 2c) resulting in quenching of UC emission at high sensitizer concentrations. However, due to the short lifetime of the annihilator’s singlet state (tens of nanoseconds), the FRET-induced quenching becomes apparent only at significantly higher concentrations, for example in PtOEP-DPA films.^37^
- Sensitizer ground-state aggregates may form at higher concentrations, leading to phosphorescence quenching. Dienel et al. reported that PtOEP aggregate formation results in a redshift of the lowest absorption band, accompanied by the appearance of a new 770 nm emission band.^38^ However, we observed no absorption or emission changes associated with aggregate formation for PtOEP concentrations up to 0.2 mM (Figure S5). Furthermore, Raišys et al. concluded that no significant quenching of PtOEP phosphorescence occurred before the onset of FRET-induced quenching at high sensitizer concentrations in PtOEP-DPA films.^37^
- Sensitizer-induced quenching of annihilator triplets via RTET reduces TET efficiency, effectively lowering *ϕ*_UC_. RTET is identified by the shortening of annihilator triplet lifetimes at high sensitizer concentrations, which also leads to an increase in *I*_th_.^39^ Annihilator triplet quenching at high sensitizer concentrations has been previously investigated in the PtOEP-DPA system.^23^ Notably, quenching occurs despite the significant energy barrier between the triplet states of PtOEP (*E*_T_ = 1.94 eV) and DPA (*E*_T_ = 1.78 eV).^40^ Additionally, a 10-fold increase in the triplet quenching rate was observed with the ZnOEP sensitizer (*E*_T_ = 1.78 eV),^41^ suggesting that a negligible energy barrier for RTET can have detrimental effects at high sensitizer concentrations.^23^ Furthermore, the triplet decay rate of the DPA annihilator was found to be unaffected by the presence of external heavy atoms at increased bromine concentrations, and also a reverse effect was observed when comparing the Zn-based sensitizer with the Pt-based sensitizer.^23^
- At high sensitizer concentrations, secondary quenching may occur due to sensitizer triplet–triplet annihilation (STTA). Arshad et al. recently reported that PtOEP in a 50 μM toluene solution exhibits a significant STTA rate constant.^42^

### Brightness of TTA-UC System

Concentration mapping of *I*_th_(50%) in the PtOEP-DPA system allowed us to identify a 10-fold excitation threshold increase at high (>0.1 mM) sensitizer concentrations (Figure 3b, see also Figure S6 for more details). This increased threshold could be related to RTET-induced annihilator triplet lifetime quenching. Independent time-resolved analysis of UC dynamics was performed to show reduction in DPA triplet lifetime from 2.4 to 0.19 ms at PtOEP concentrations ranging from 10 to 100 μM, respectively (Figures S7b and S7d). This is consistent with previous reports showing that annihilator triplets are quenched via triplet energy transfer to the sensitizer.^23^ Additionally, delayed PtOEP phosphorescence with a lifetime matching that of the DPA triplet was observed (Figures S7a and S7c), further confirming the presence of RTET in the PtOEP-DPA system.

Maximum potential brightness (*ϕ*_UC_ × *A*_max_) is a key parameter derived from the automated concentration mapping, representing the attainable UC intensity per incident photon at the sensitizer’s absorbance peak. Essentially, it is the emitted light intensity measured before relative quantum yield correction but with adjustments for outcoupling, incoupling, and the maximum available absorbance at the given sensitizer concentration. For applications such as solar energy conversion, 3D printing, and drug activation, the efficiency metric of interest is based on the number of incident photons rather than the number of absorbed photons. A decrease of *ϕ*_UC_ at high sensitizer concentrations can be tolerated in exchange for higher photon absorption. However, many applications also aim to keep sensitizer and annihilator concentrations as low as possible to save expensive materials. Therefore, this metric should enable differentiation between compositions for the highest UC emission output at the lowest sensitizer and annihilator concentrations.

Mapping *ϕ*_UC_ × *A*_max_ shows that the UC emission output steadily increases with sensitizer and annihilator concentrations, reaching maximum values at sensitizer concentrations beyond 0.1 mM (Figure 3b). This observation aligns with the low losses observed at high sensitizer concentrations. Intriguingly, at annihilator concentrations above 0.5 mM, the emitted photons over incident photons exhibit marginal growth, which is associated with a plateau of *ϕ*_UC_ shown in Figure 3a. On the other hand, O’Dea et al. employed the PtOEP-DPA upconverting system for DLP 3D printing,^8^ where a DPA annihilator concentration of up to 5 mM was used to overcome oxygen quenching under ambient conditions.

### Screening of New TTA-UC Systems

**Figure 4:**
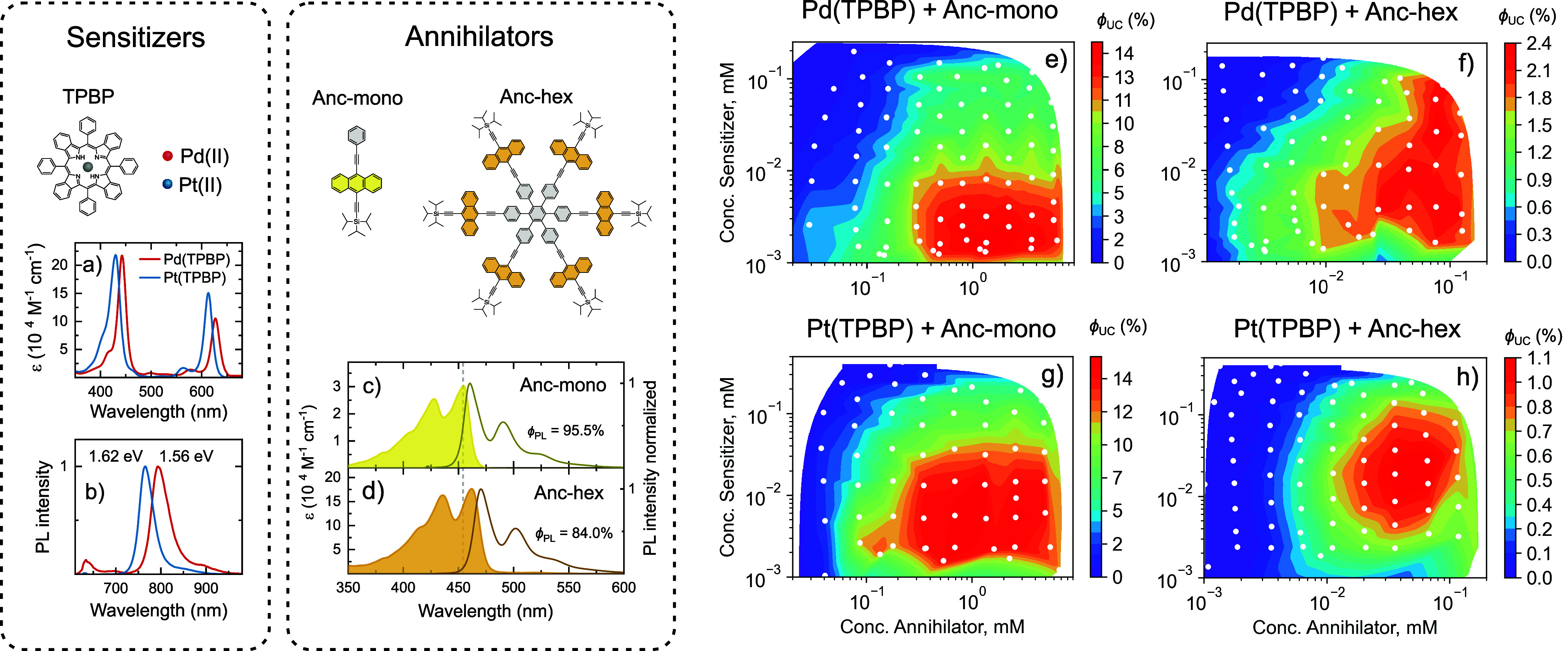
Concentration mapping of the upconversion quantum yield (*ϕ*_UC_) for different NIR-absorbing sensitizer and anthracene-based annihilator combinations. (a) Absorbance and (b) photoluminescence spectra of TPBP-based sensitizers. Lowest triplet energy levels were estimated from peaks of phosphorescence spectrum. Annihilator absorbance and emission spectra of (c) Anc-mono in 10 μM and (d) Anc-hex in 1 μM toluene solution. Photoluminescence quantum yields (*ϕ*_PL_) determined by the integrating sphere method in the same solutions are indicated. (e and f) Concentration maps of *ϕ*_UC_ (50% maximum) for different sensitizer–annihilator combinations.

As described above in detail, our automated TTA-UC platform provides unprecedented ability to rapidly screen multiple parameters in sensitizer–annihilator systems and visualize key parameters. We examined the scope of this automated system on a new through-space coupled triisopropyl((12-(phenylethynyl)anthracene-5-yl)ethynyl)silane (TIPS-anthracene-phenyl, Anc-mono) based hexamer (Anc-hex, Figure 4), designed to explore how annihilator unit density affects TTA efficiency. The design of the hexamer was inspired by the successful TTA-UC annihilator TIPS-anthracene,^43,44^ reported for its high *ϕ*_UC_ associated with a large spin-statistical factor.^29^ Additionally, recent studies have shown that analogous through-space coupled tetracene hexamers exhibited quantitative (197%) triplet pair formation efficiency via singlet fission,^45^ indicating the potential of hexamers to support multiple triplet excitations.

TTA-UC parameters of Anc-hex were compared with those of its constituent monomer Anc-mono (Figure 4) and TIPS-anthracene (Figure S8). Similarly to TIPS-anthracene, in diluted solutions both Anc-mono and Anc-hex displayed high fluorescence quantum yields of 95.5% and 84.0%, respectively. Anc-hex shows exactly 6-fold higher molar absorption coefficient compared to Anc-mono, indicating weak interchromophore interactions in hexamer (Figure 4d). Notably, full concentration mapping of TIPS-anthracene paired with palladium(II) meso-tetraphenyltetrabenzoporphyrin (Pd(TPBP)) sensitizer resulted in almost identical maximum *ϕ*_UC_ values of 27% as reported recently by Nishimura et al. in the same concentration region,^29^ further validating the accuracy of the automated TTA-UC concentration screening platform.

As sensitizers, we have selected two common heavy metal containing porphyrins: Pd(TPBP) and Pt(TPBP) (Figure 4) possessing Q-bands within the 633 nm laser excitation region. Slight variation in triplet energy level of 1.56 and 1.62 eV for Pd(TPBP) and Pt(TPBP), respectively, may influence TET as well as RTET dynamics. In our screening experiments, all sensitizer–annihilator combinations resulted in significant quenching of sensitizer phosphorescence with increasing annihilator concentration, which is an indication of an efficient TET approaching unity at practically relevant annihilator concentrations (Figures S12–S15c). TET efficiency was estimated based on intrinsic phosphorescence quantum yields, which equals to 9% for Pd(TPBP)^27^ and 70% for Pt(TPBP).^46^

To evaluate the capacity of the annihilators to receive triplet energy from the sensitizer, the TET efficiency for all sensitizer–annihilator combinations was plotted as a function of the annihilator concentration (Figure S14). The higher TET efficiency observed at lower annihilator concentrations for the hexamer, compared to the monomer, suggests that the hexamer is more effective at quenching sensitizer triplets. This indicates that, under conditions of statistically similar molecular collision rates between sensitizer and annihilator molecules, an increased number of accepting units (anthracenes) enhances the probability of TET.

To account for the larger number of annihilator units in the hexamer, TET efficiency as a function of effective annihilator concentration (calculated by multiplying the molar hexamer concentration by 6) was plotted in Figure S14. When paired with the higher triplet energy Pt(TPBP) sensitizer, the effective TET efficiency of Anc-hex was nearly identical with that of Anc-mono. This indicates that cooperative behavior is negligible, as the TET efficiency scales directly with the annihilator density. However, when the hexamer was paired with the lower triplet energy sensitizer Pd(TPBP), the effective TET efficiency was reduced compared to that of the monomer and TIPS-anthracene. This reduction can likely be attributed to the higher triplet energy level of the hexamer relative to that of the monomer. Supporting this, DFT calculations of triplet energies showed the lowest triplet state of Anc-hex to be approximately 70 and 130 meV higher than TIPS-Anc and Anc-mono, respectively (see Figure S16 and Section S6 for details on the theoretical calculations).

Concentration mapping of *ϕ*_UC_ for all sensitizer–annihilator combinations is shown in Figures 4e–4h. The maximum achieved *ϕ*_UC_ values were 14.7%, 14.5%, 2.7%, and 1.1% for Pd(TPBP):Anc-mono, Pt(TPBP):Anc-mono, Pd(TPBP):Anc-hex, and Pt(TPBP):Anc-hex sensitizer–annihilator combinations, respectively. The variation of *ϕ*_UC_ in hexamer TTA-UC systems was found to be caused by formation of ground-state aggregates at concentrations above 0.1 mM (for more details on aggregate formation see Section S7). Nevertheless, aggregate formation could not account for the large decrease in *ϕ*_UC_ values observed in Anc-hec compared to Anc-mono.

Using *ϕ*_UC_ and *ϕ*_TET_ obtained from automated screening, along with *ϕ*_PL_ from independent integrating sphere measurements, spin-statistical factors were estimated according to eq 1 (see Table S1 and Section S8). The calculated spin-statistical factors for TIPS-anthracene, Anc-mono, and Anc-hex were 59.9%, 29.9%, and 5.0%, respectively. The 2-fold decrease in the spin-statistical factor for Anc-mono compared to TIPS-anthracene aligns with recent findings that TIPS-acetylene groups enhance coupling between triplet and singlet states, thereby increasing the *f* factor.^47^ However, the nearly 6-fold reduction (29.9% vs 5.0%) of the spin-statistical factor in Anc-hex compared to its constituent monomer suggests that the annihilator units containing TIPS-acetylene groups are decoupled in the hexamer. Furthermore, this indicates that the spin-statistical factor is influenced by the spatial distribution and orientation of annihilator units; a high density of such units, if arranged in nonoptimal positions, results in a lower probability of TTA compared to independent annihilator monomers, which can randomly align during annihilator–annihilator collisions in solution.^41^

Despite the clear negative effects of suppressed spin-statistical factor and annihilator aggregation limiting the maximum *ϕ*_UC_ of the hexamer-based TTA-UC systems, evidence of reduced triplet quenching at high sensitizer concentrations was observed. Concentration mapping of Anc-hex showed that *ϕ*_UC_ was only weakly quenched as the Pd(TPBP) sensitizer concentration increased, compared to Anc-mono at equivalent sensitizer concentrations (Figures 4e and 4f and Figure S19). A similar, though less pronounced, reduction in sensitizer quenching was observed for the hexamer paired with the Pt(TPBP) sensitizer (Figure 4h and Figure S19). Both Anc-mono and Anc-hex reached maximum *ϕ*_UC_ values at annihilator concentrations of 0.3–0.5 and 0.04–0.08 mM, respectively, which are comparable when normalized to effective anthracene unit concentration. This suggests that using a coupled annihilator hexamer may help mitigate sensitizer-induced quenching.

With increasing sensitizer concentration, *ϕ*_UC_ of Anc-mono was significantly quenched (up to 5-fold) when paired with Pd(TPBP) and Pt(TPBP) sensitizers (Figures 4e and 4g, respectively). Notably, Pd(TPBP) induced a more pronounced quenching even at moderate sensitizer concentrations of 10–100 μM. Losses at high sensitizer concentrations may occur through various mechanisms, such as FRET from the annihilator singlet to the sensitizer, sensitizer aggregation, sensitizer triplet self-quenching (STTA), or sensitizer-induced annihilator triplet quenching (RTET).

FRET-induced losses can be identified by reduction of the annihilator *ϕ*_PL_ or fluorescence lifetime at high sensitizer concentration. Both Pd(TPBP) and Pt(TPBP) exhibited moderate FRET-induced losses resulting in approximately 17% and 20% reduction of Anc-mono fluorescence lifetime, respectively, at a sensitizer concentration of 0.2 mM (see Figure S20). This also agrees with minimal overlap between Anc-mono annihilator emission and sensitizer absorption (Figures 4a and 4c). Contrary to the observed drop of *ϕ*_UC_, FRET-induced losses were slightly higher for the Pt(TPBP) sensitizer. Therefore, the majority of *ϕ*_UC_ losses at high sensitizer concentration can be attributed to other factors discussed below.

To identify the effects of sensitizer aggregation, phosphorescence decay dynamics of Pd(TPBP) were measured as a function of concentration. The lifetime gradually reduced from 285 to 104 μs when the concentrations were changed from 5 to 100 μM (Figure S21). Almost 3-fold lifetime reduction indicated significant triplet quenching, which indicates that it is the main mechanism for reduction of *ϕ*_UC_ at high sensitizer concentrations. Similarly, the lifetime of Pt(TPBP) was previously shown to drop from 20 to 6 μs for concentrations of 20 μM and 1 mM, respectively.^29^

Significant dynamic quenching due to STTA was also observed at increased excitation densities with high Pd(TPBP) concentrations (Figures S21a–S21c). Additionally, a delayed fluorescence signal at 640 nm was detected in Pd(TPBP) solutions, providing direct evidence of singlet state formation via STTA (Figure S21d). Similarly, STTA was observed in concentrated Pt(TPBP) sensitizer solutions, where the estimated STTA rate constant was more than twice that of the PtOEP sensitizer.^42^

Another prominent quenching mechanism at high sensitizer concentrations is sensitizer-induced annihilator triplet quenching via RTET, which highlights differences in the *ϕ*_UC_ sensitizer-induced quenching between Pd(TPBP) and Pt(TPBP) sensitizers. The increase in the *I*_th_(50%) value beyond a 40 μM Pd(TPBP) sensitizer concentration is a strong indicator of sensitizer-induced quenching of the annihilator triplet (Figure S22c). In contrast, no increase in *I*_th_(50%) was observed with increasing Pt(TPBP) concentration (Figure S22d).

The upconverted emission lifetime (τ_UC_) of Anc-mono was decreased significantly from 66 to 42 μs as the Pd(TPBP) sensitizer concentration increased from 3 μM to 0.2 mM (Figure S23a). In contrast, a much longer τ_UC_ of 136 μs was observed when 2 mM Anc-mono was paired with 0.2 mM Pt(TPBP) sensitizer (Figure S23b). This difference in quenching rates cannot be attributed to the external heavy atom effect as the heavier Pt-based sensitizer induced weaker quenching. The higher quenching rate with Pd(TPBP) may result from a lower energy barrier for RTET compared to Pt(TPBP) (Figure S16). Notably, even a small increase in the energy barrier of 60 meV is expected to reduce the RTET losses, particularly at high sensitizer concentrations. Although the exact energy barrier for the Anc-mono is difficult to determine, structurally similar TIPS-anthracene (*T*_1_ = 1.37 eV)^48^ has energy barriers of 190 and 250 meV when paired with Pd(TPBP) and Pt(TPBP), respectively.

## Conclusion

We have successfully built and tested an automated screening platform for TTA-UC measurements that accelerates workflow (over 100 sensitizer–annihilator concentration scans in approximately 2 h) and conserves materials and solvents (down to 0.3 mL per scan). Main TTA-UC parameters of known sensitizer–annihilator pairs PtOEP:DPA and PtTPBP:TIPS-anthracene were concentration-mapped to visualize optimal concentrations and identify loss pathways. Beyond its clear importance for TTA-UC, the automated screening method demonstrates how multicomponent photochemical systems can be optimized in a simple and accessible way.

In terms of maximum UC quantum yield, screened molecular combinations suggested optimal sensitizer and annihilator concentrations around 10 μM and 1 mM, respectively. Visualization revealed that the optimal annihilator concentration is constrained by the efficiency of triplet energy transfer from the sensitizer and the oxygen concentration in the sample. For practical applications, exceeding the annihilator concentration beyond the point of maximum upconversion quantum yield provides no additional benefit as the emission output will depend solely on the number of photons absorbed.

Optimizing the sensitizer concentration is more complex and depends on four key parameters: the overlap between annihilator emission and sensitizer absorption, the relative triplet energy compared to the annihilator triplet, and the sensitizer’s susceptibility for aggregation and triplet–triplet annihilation. A large spectral overlap can cause UC emission losses due to energy back-transfer to the sensitizer. However, most studied metal–ligand sensitizers possess a transparency window for the used annihilators, leading to relatively small losses in solution. The energy barrier for reverse triplet transfer between the sensitizer and annihilator helps to prevent annihilator triplet quenching, even at high sensitizer concentrations. An increase in the sensitizer–annihilator energy barrier by as little as 60 meV has been shown to significantly reduce the annihilator triplet quenching and minimize the excitation threshold intensity. Our analysis of various sensitizer–annihilator combinations revealed that significant losses at high sensitizer concentrations (up to 5-fold reduction of maximum upconversion quantum yield at 0.2 mM sensitizer concentration) are primarily due to sensitizer triplet self-quenching via aggregation and sensitizer-TTA. This was found to significantly affect losses in common tetraphenyltetrabenzoporphyrin-based sensitizers.

Additionally, automated concentration mapping was performed on a new hexameric annihilator containing TIPS-acetylene-anthracene units paired with common TPBP-based sensitizers. The estimated spin-statistical factor for the hexamer, at 4.6%, was significantly lower than the 29.9% estimated for the constituent monomer. This suggests unfavorable spatial alignment of the annihilator units for intermolecular or intramolecular TTA and a lack of electronic coupling in the through-space coupled anthracene hexamers.

## Materials and Methods

Full description of automated TTA-UC equipment, materials, and methods can be found in the Supporting Information. A Python program for the automated TTA-UC setup control, data analysis program, and data samples can be found in the code repository (https://github.com/Elholm/KMP-Group).

## Supporting Information Available

Description of materials and methods, Figures S1–S29, and description of new material synthesis procedure (PDF)
Transparent Peer Review report available (PDF)
