# Supplementary material for: Automated Research Platform for Development of Triplet–Triplet Annihilation Photon Upconversion Systems
Source: ACS Cent Sci. 2025 Feb 21;11(3):413–21. doi: 10.1021/acscentsci.4c02059 (PMC11950846; doi:10.1021/acscentsci.4c02059)
Supplement: Supplementary file 1 — oc4c02059_si_001.pdf [file oc4c02059_si_001.pdf]

Supporting information

Automated Research Platform for Development of Triplet-Triplet Annihilation Photon Upconversion Systems

*Paulius Baronas, Justas Lekavičius, Maciej Majdecki, Jacob E. Lynge, Karolis Kazlauskas, Przemysław Gaweł and Kasper Moth-Poulsen**

1. Materials and Methods

Materials. Synthesis of new compounds **Anc-mono** and **Anc-hex** was performed in Institute of Organic Chemistry, Polish Academy of Sciences. Full synthesis procedure, materials, general methods and NMR spectra are presented in Section S10. A 532nm absorbing sensitizer platinum(II) octaethylporphyrin (**PtOEP**) was purchased from Sigma-Aldrich. A 633nm absorbing sensitizers Pd(II) meso-tetraphenyltetrabenzoporphyrin (**Pd[TPBP]**) and Pt(II) meso-tetraphenyltetrabenzoporphyrin (**Pd[TPBP]**) were purchased from Frontier Specialty Chemicals. Commercial annihilators 9,10- diphenylanthracene (**DPA**) and 9,10-Bis[(triisopropylsilyl)ethynyl]anthracene (**TIPS-anthracene**) were purchased from Sigma-Aldrich.

Photon upconversion and phosphorescence transients. Time-resolved measurements were performed in Institute of Photonics and Nanotechnology, Vilnius University. Samples for time-resolved analysis were prepared in a glovebox with known O_2 concentration of 2 ppm resulting in negligible oxygen concentration in tested solutions. UC and PH transients of the samples were measured using a time-gated intensified CCD camera New iStar DH340T (Andor) coupled to a spectrograph SR-303i (Shamrock) serving as the detector. In these experiments, a nanosecond YAG:Nd3+ laser NT 242 (Ekspla) equipped with an optical parametric oscillator (repetition rates 1 kHz and 100 Hz, excitation wavelengths 532 nm and 640 nm for PtOEP and for PdTPBP and PtTPBP sensitizers) was used for sample excitation. UC transients measured at low excitation power density were approximated by a single exponential function to extract UC lifetime using the Lifetime fitting procedure is described elsewhere [1].

2. Automated TTAUC measurement platform

Full automated TTAUC screening platform is displayed in Figure S1a. Dilutions were performed by three pumps, two programable syringe pumps (KDS LEGATO 110) with 10 ml syringes (Hamilton 010 TLL) for dispensing concentrated sensitizer and annihilator solutions and peristaltic pump (Vapourtec SF-10) for solvent. The highest achievable dilution ratio (or the concentration dynamic range) is roughly determined by pump precision at low flow rate range and by total flow rate that is used to create dilution. The lowest flow rate we chose to operate was 1 µl/min given the high precision of syringe pumps used to dispense sensitizer and emitter solutions. Mixing is performed at constant total 1000 ul/min flow rate, that would result in roughly 1:1000 dilution ratio for both sensitizer and emitter solutions. Predetermined dilutions

were generated at portions of 300 µl, which is significantly larger than 50 ul internal volume of the flow cell to minimize the effects of dispersion inside the system.

For degassing of premixed solution in flow we used a 925 µl degassing unit (IDEX) based on oxygen permeable membrane tube inside vacuum chamber. A minimum of 0.4 mbar pressure inside the degassing chamber was reached using a dual-stage rotary vane mechanical vacuum pump (Edwards RV3). The vacuum pressure was measured with an electronic vacuum gauge (Vacuubrand Vacuu-View extended). Degassing of continuously flowing solution was described in detail in our previous paper [2]. We have found that some oxygen reabsorption was evident in the fluoropolymer tubing connecting degassing unit to the flow cell resulting in drop of potential degassing efficiency. In the current work, to minimize the effects of oxygen reabsorption, we used stainless steel tubing (length 20 cm, I.D. 0.5 mm, IDEX) between degassing unit and flow cell. Testing with same 5 µM PtOEP in toluene solution as in previous paper we observed 270 times increase of phosphorescence emission intensity in degassed solution compared to ambient air saturated solution. According to Henry's equation and Henry's constant of 1050 bar for oxygen solubility in toluene [3], ambient oxygen molar concentration is approximately 10 mM in toluene. Based on Stern–Volmer analysis, maximum degassing efficiency with stainless steel tubing results in 4.8 µM of oxygen in toluene solutions. Considering 0.4 mbar vacuum in degassing chamber, Henry's equation gives theoretical 3.6 µM oxygen concentration in toluene. Good agreement between theoretical and measured values suggests negligible oxygen reabsorption in stainless steel tubing connecting flow cell to degassing unit.

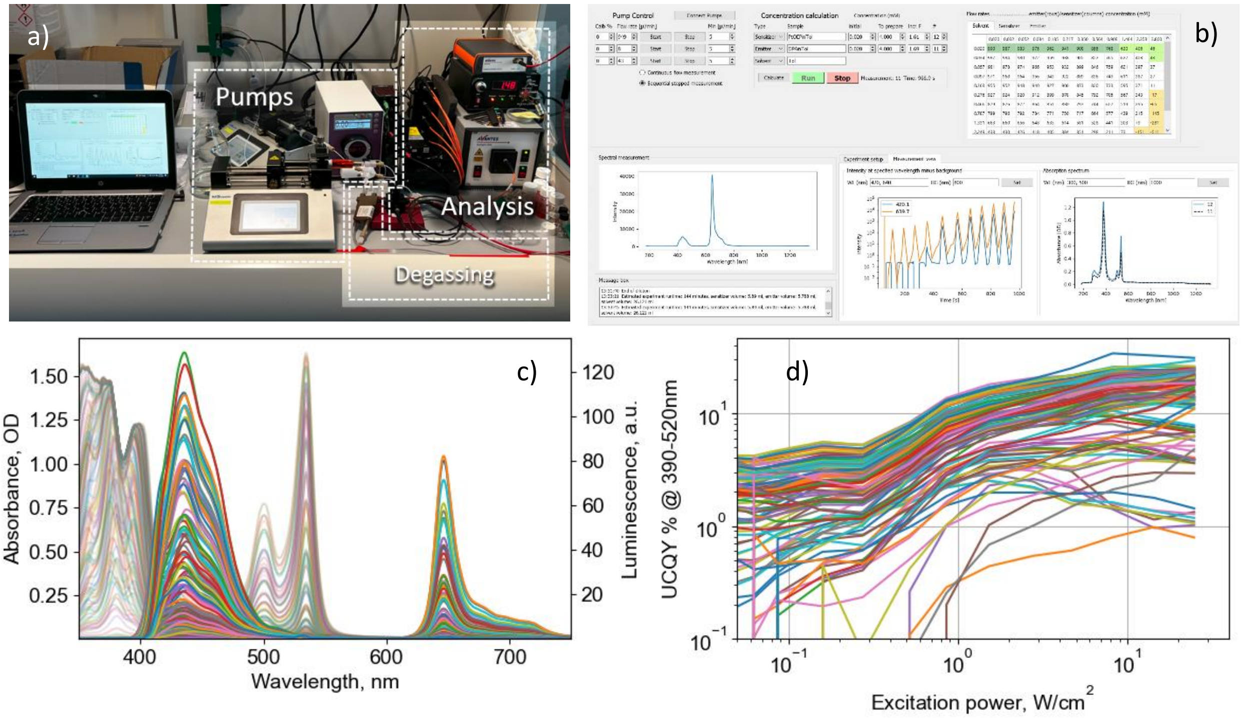

Figure S1. (a) Picture of automated TTA-UC platform with main systems identified. (b) Screenshot of TTA-UC platform control software. Over 165 minutes TTAUC platform collected 122 concentration scans identified by unique (c) absorption and emission spectra and (d) at each concentration emission spectra were measured at 16 excitation powers.

Also, system was operated employing “mix-stop-measure” sequence. After dilution was generated, the flow is stopped for a period of time allowing oxygen concentration in solution to reach equilibrium with oxygen in the degassing unit. This time duration is determined by later stage of spectroscopic characterization of TTA-UC properties (roughly 2-3 minutes). The following mixing step is pushing previous dilutions towards flow cell for characterization at 1000 ul/min flow rate to avoid oxygen reabsorption. We found that “mix-stop-measure” sequence is superior to continuous operation at 300 ul/min flow rate due to lower residual oxygen concentration in measured solution, less material required for a single dilution and unlimited time for measurement of TTA-UC characteristics.

Spectroscopic characterization was performed in 2-way quartz flow cell (Hellma 176.762-QS). The minimal 1.5 mm path length was selected for absorbance measurements of high concentration solutions. The flow cell holder (Avantes CUV-ALL/UV/VIS) was coupled to fiber-optic-switch (Avantes) that could switch between broad band deuterium-halogen light source (Avantes AvaLight-D(H)-S) for absorbance measurement and laser excitation. To cover broad excitation spectrum three continuous wave (CW) lasers of 532 nm (RLTMGL-532-200-3), 633 nm (RLTMDL-633L-80-2) and 730 nm (RLTMDL-730L-30-2) were integrated. Laser power modulation was achieved by blocking type variable optical attenuator (OZ Optics DD-100-11-532/730-105/125-QM) providing up to 50db attenuation dynamic range that is sufficient to detect excitation threshold for most TTA-UC systems. The spectrometer (Avantes AvaSpec-ULS2048CL-EVO-RS-UA) recording sequence is setup to record absorbance spectrum followed by recording luminescence spectrum at each excitation power in increasing order (Figures S1 c and d). The luminescence detection was performed by dynamically changing exposure to avoid intensity saturation.

A Python program with user graphical interface (Figures S1b) was written to control the pump flow rates, laser excitation power and detect absorbance and emission changes. Python based data analysis program was written to determine concentrations from measured absorbance, determine upconversion quantum yields, triplet energy transfer efficiencies, excitation threshold. To visualize the 3D data with non-equivalent X and Y coordinates—corresponding to the measured sensitizer and annihilator concentrations— the data was first interpolated onto a fine grid (1000x1000 points). To minimize artifacts introduced during interpolation a Gaussian filter (σ=5, order=0) was applied to smooth the data. The resulting smoothed data was used to generate contour plots. The color maps represent interpolated values between the lowest and highest measured points in the XY coordinates, no extrapolation was employed. The data acquisition and analysis programs are published in the code repository (https://github.com/Elholm/KMP-Group).

3. Determination of emission quantum yields

Emission quantum yields were measured using relative method and standard fluorescence dyes [4]. For 532 nm laser excitation Rhodamine B in Ethanol was used as fluorescent standard dye (ϕ_{PL} = 70 %) [5], [6]. For 633 nm laser excitation Cresyl Violet in Ethanol was used as standard fluorescent dye (ϕ_{PL} = 58 %) [6]. Figure S2 shows a procedure of determining calibration constant (m_s) for Cresyl violet. Multiple low absorbance (OD < 0.1) samples were measured to find m_s that corresponded to slope of linear fit of integrated emission intensity versus absorbance at excitation wavelength. In the calculation of emission quantum yield, difference of refractive index (*n*) of standard and sample was considered.

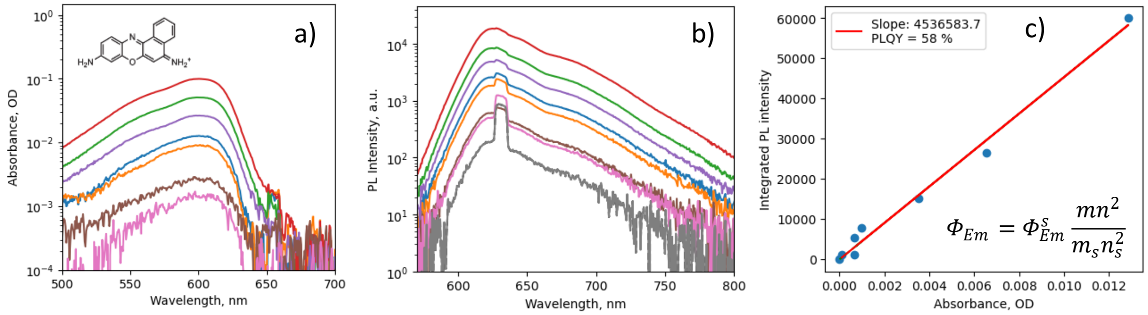

Figure S2. Relative method for determination of emission quantum yield. (a) Absorption and (b) emission spectra spectra of Cresyl Violet standard in ethanol (Φ_s = 58%) at different concentrations. (c) Linear fit of integrated emission versus absorbance, with slope (m) indicated. Excitation wavelength was set to 633 nm.

4. Emission intensity correction at strong sensitizer absorbance

The emission quantum yields recorded in the system were exhibiting abnormal reduction at high sensitizer concentrations that was not related to any quenching mechanism. This reduction in emission intensity was associated with poor optical incoupling determined by how much light is collected at different absorption length of excitation wavelength. In an automated TTAUC system it is a critical correction that could lead to underestimation of ϕ_{UC} at high sensitizer concentrations. The calibration was performed with fluorescent standard dye showing high absorbance at excitation wavelength and no concentration quenching in the measured range. The decrease of Rhodamine B emission quantum yield at strong absorption (OD > 0.1) of 532 nm light is presented in Figure S3. This effect cannot be associated with concentration induced quenching due to relatively low concentrations (10^{-6} – 10^{-4} M) of Rhodamine B solutions [5].Therefore, the incoupling correction curve was applied to the concentration maps to correct the emission intensity and ϕ_{UC} values at high sensitizer concentrations. We have also performed a measurement of phosphorescence quantum yield (ϕ_{Phos}) of degassed PdTPBP toluene solution versus concentration. 633 nm laser excitation power was set as low as possible to detect signal and avoid excitation density induced annihilation. Nevertheless, significant reduction of ϕ_{Phos} was observed due to triplet-triplet annihilation occurring at elevated concentrations. This shows that incoupling correction must be performed with fluorescent materials and low laser excitation densities.

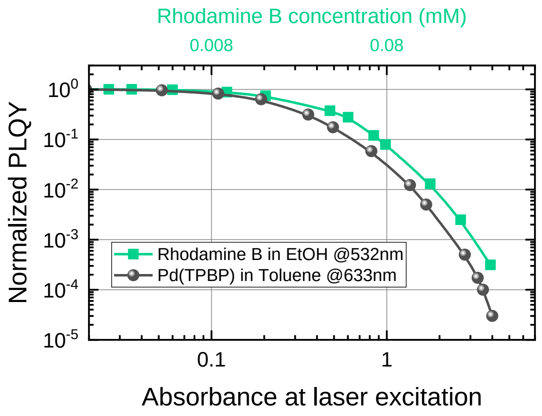

Figure S3. Normalized photoluminescence quantum yield vs absorbance at excitation wavelength. Was measured as Rhodamine B emission quantum yield was used for optical incoupling efficiency correction due to low concentration dependence below 1 mM concentration. For reference, phosphorescence quantum yield of degassed Pd(TPBP) solutions showed more significant reduction at same absorbance values in same flow cell suggesting concentration related quenching.

5. Determination of triplet energy transfer quantum yield

The ϕ_{Phos} of 10% recorded at 10^{-5} M annihilator concentration in PtOEP-DPA solution was around four times lower compared to ϕ_{Phos} ~ 40% of PtOEP in solution [7]. If all the loss of ϕ_{Phos} contributed to energy transfer to annihilator, this would correspond to ϕ_{TET} = 75%. Unfortunately, residual oxygen in mixture can also act as quencher of PtOEP triplets, resulting in competing TET and oxygen quenching pathways,which explains a significant drop of ϕ_{UC} at low annihilator concentrations below 0.1 mM.

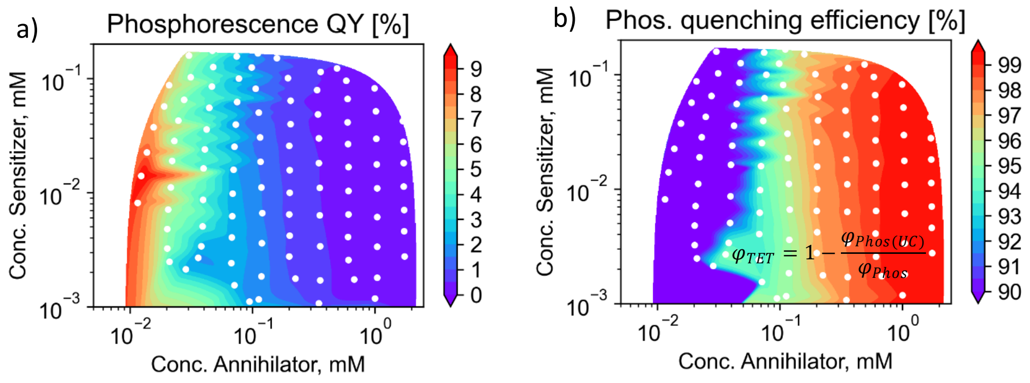

Figure S4. Concentration map of phosphorescence parameters of PtOEP-DPA system. (a) phosphorecence quantum yield for emission integrated in 630-800 nm region. (b) phosphorescence quenching efficiency, where phosphorescence quantum yield of PtOEP sensitizer without annihilator is set to 40 %. Excitation wavelength was set to 532 nm.

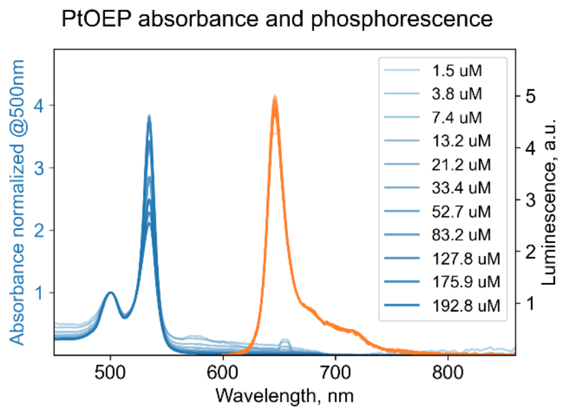

Figure S5. Normalized absorbance and phosphorescence spectra of PtOEP in toluene at different concentrations. Absorbance measurements were performed in 1.5 mm path cell. Due to strong light filtering in 520-540 nm region absorbance peak was distorted and underestimated at increased concentrations. Phosphorescence spectra were normalized at 670 nm.

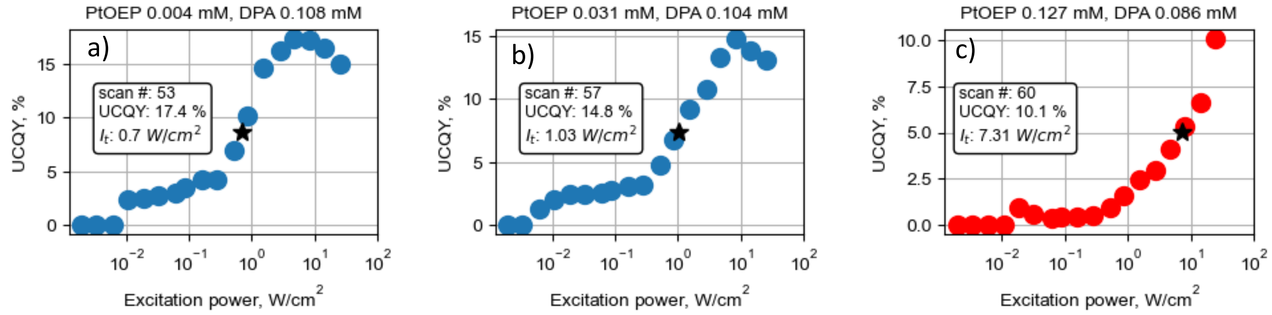

Figure S6. Upconversion quantum yield (UCQY) versus excitation power in the increasing order of PtOEP sensitizer concentration (a-c). DPA annihilator concentration was kept at constant 0.1 mM. Red circles indicate that UCQY is not reaching maximum value at highest excitation density due to high excitation threshold (I_t) indicated by star symbol.

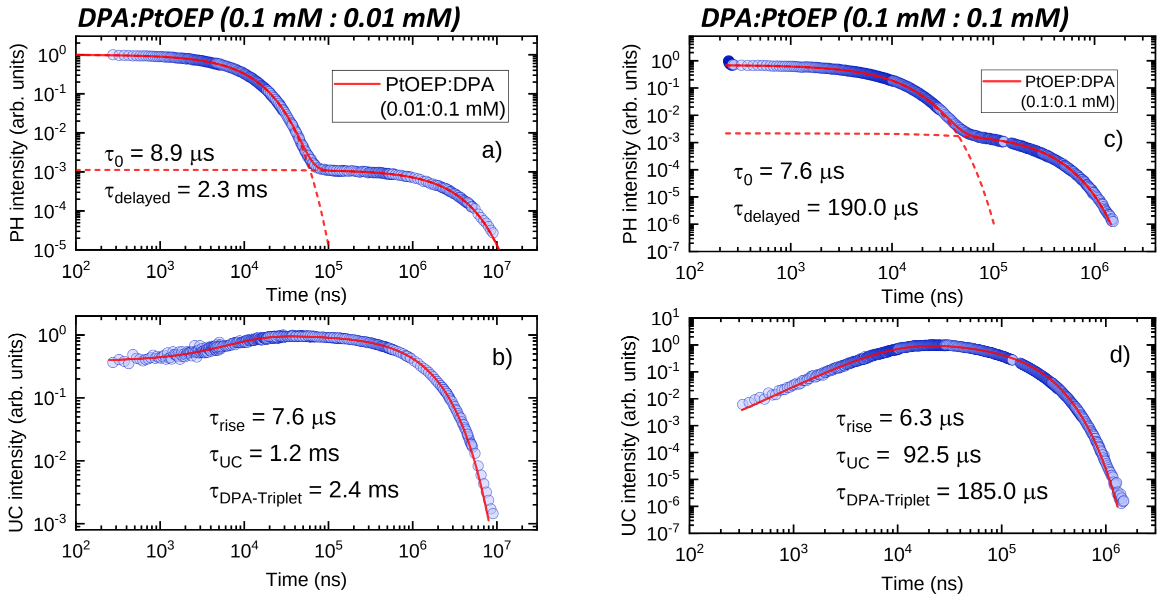

Figure S7. (a,c) Phosphorescence and (b,d) upconverted emission transients recorded in 100 µM DPA annihilator with 10 µM and 100 µM PtOEP sensitizer concentration. Transients represent integral of emission spectrum (400-460 nm for DPA UC emission and 625-675 nm for PtOEP phosphorescence). Lifetime fitting procedure is described elsewhere.[1] The two fitted components in (a,c) represent prompt (τ_0) and delayed ($\tau_{delayed}$) phosphorescence. The two fitted components in (b,d) represent risetime (τ_{rise}) and decay (τ_{UC}) of upconversion emission. DPA triplet lifetime is estimated as double of τ_{UC} due to two triplets required for the TTA event. Reverse triplet energy transfer (RTET) was observed as reduction of DPA lifetime and appearance of stronger delayed phosphorescence component at high sensitizer concentration. Laser parameters: 532 nm, E = 2.5 µJ, A = 11 mm^2.

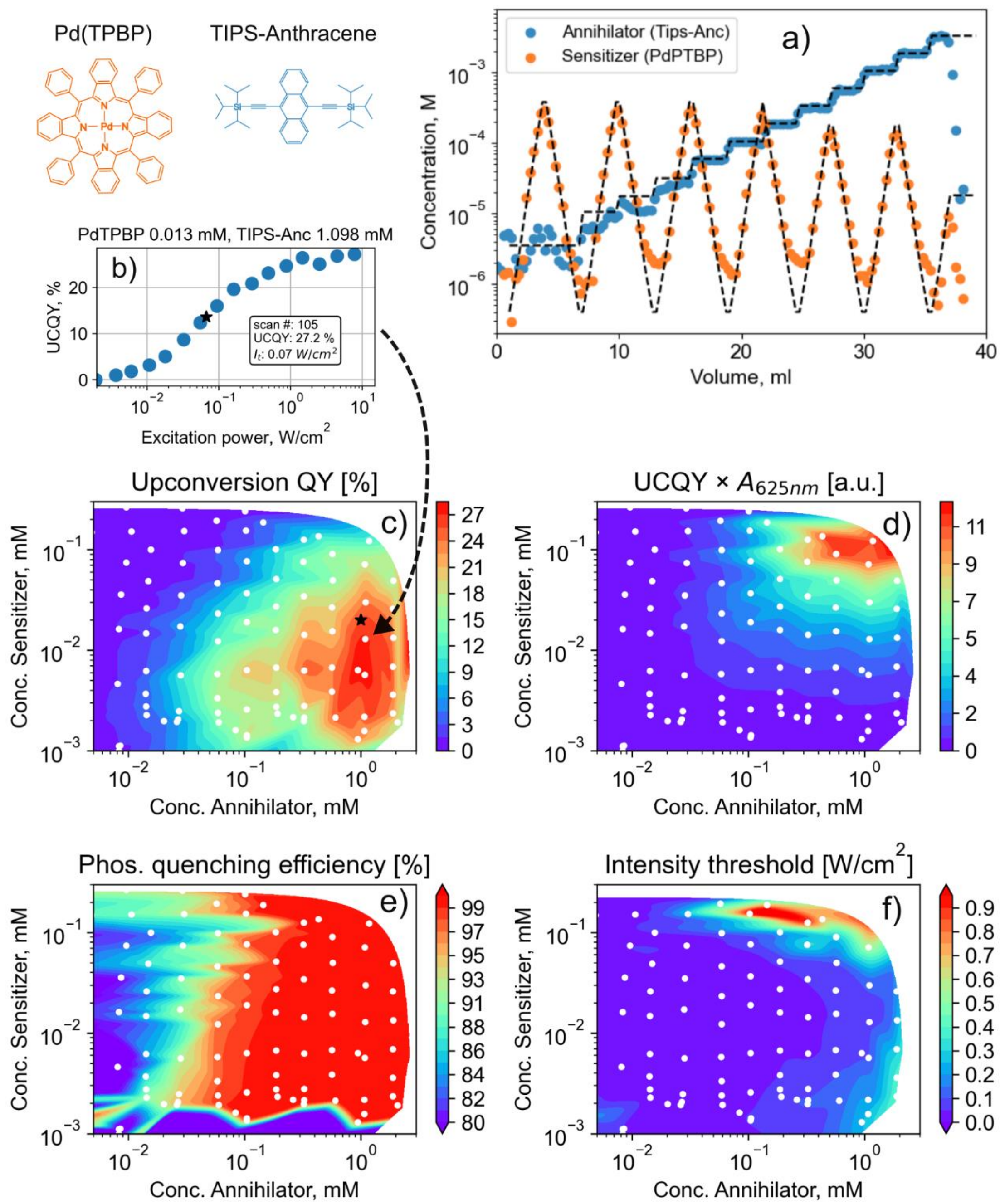

Figure S8. Full automated characterization of TIPS-Anthracene annihilator and Pd(TPBP) sensitizer system TTAUC. (a)Estimated sensitizer and annihilator concentrations from absorbance measurements. (b) Excitation power dependence of UCQY at specified sensitizer-annihilator concentrations. (c) Concentration map of upconversion quantum yield. Black star indicates UCQY value of 27% reported in the literature.[8] *(d) Concentration map of potential brightness corresponding to upconverted emission output per incident photons at peak sensitizer absorbance of 625 nm. (e) Concentration map of triplet energy quenching efficiency calculated with intrinsic Pd(TPBP) phosphorescence quantum yield of 9%.*[9] *(f) Concentration map of excitation intensity threshold (*I_{th}*(50%)) in total incident power units. White dots indicate 127 measured concentration combinations.*

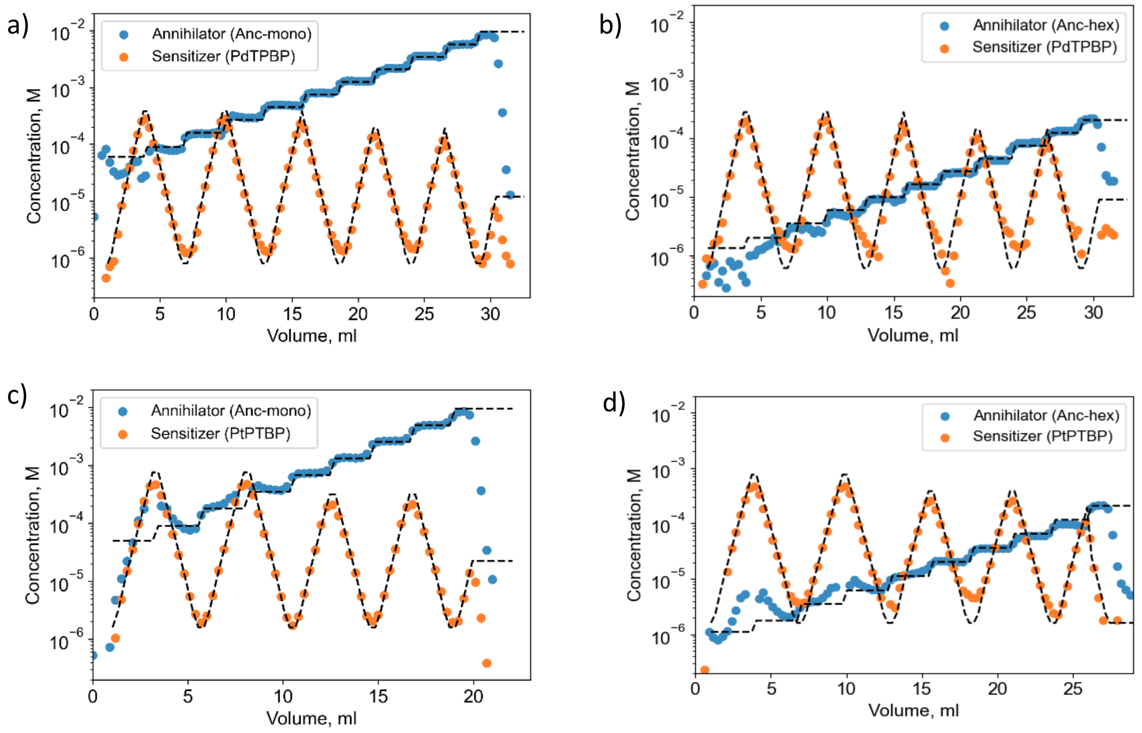

Figure S9. Estimated sensitizer (Pd(TPBP) and Pt(TPBP)) and annihilator (Anc-mono and Anc-hex) concentrations from absorbance measurements in automated TTAUC system and using predetermined molar extinction coefficients. For precision, molar extinction coefficients were determined independently in diluted solutions of low molar concentration (1-10 µM).

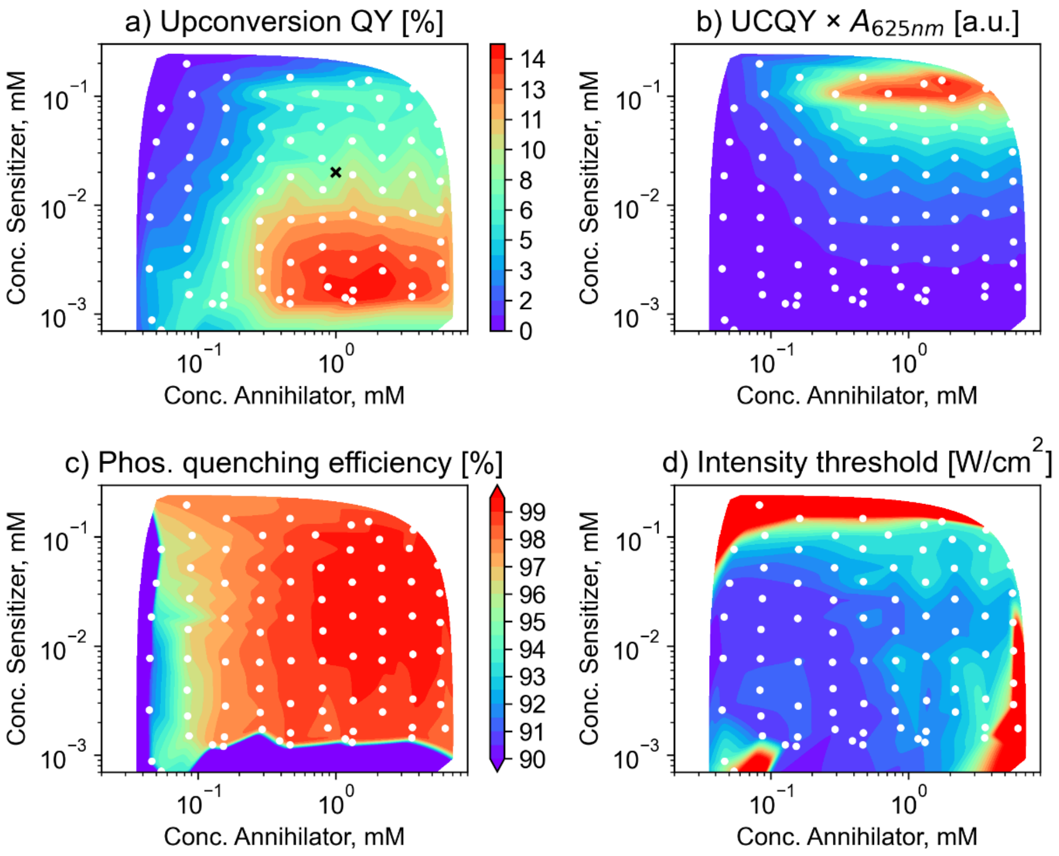

Figure S10. Concentration mapping of main TTAUC parameters in Pd(TPBP) sensitizer and Anc-mono annihilator system. (a) Upconversion quantum yield. Black cross indicates position at 20 µM sensitizer and 1 mM annihilator concentration. (b) Potential brightness corresponding to upconverted emission output per incident photons at peak sensitizer absorbance of 625 nm. (c) Phosphorescence quenching efficiency calculated with 9% intrinsic phosphorescence efficiency of Pd(TPBP). (d) Excitation intensity threshold power for 633 nm excitation determined at half upconversion efficiency. Concentrations were estimated from absorbance measurements (Figure S11a).

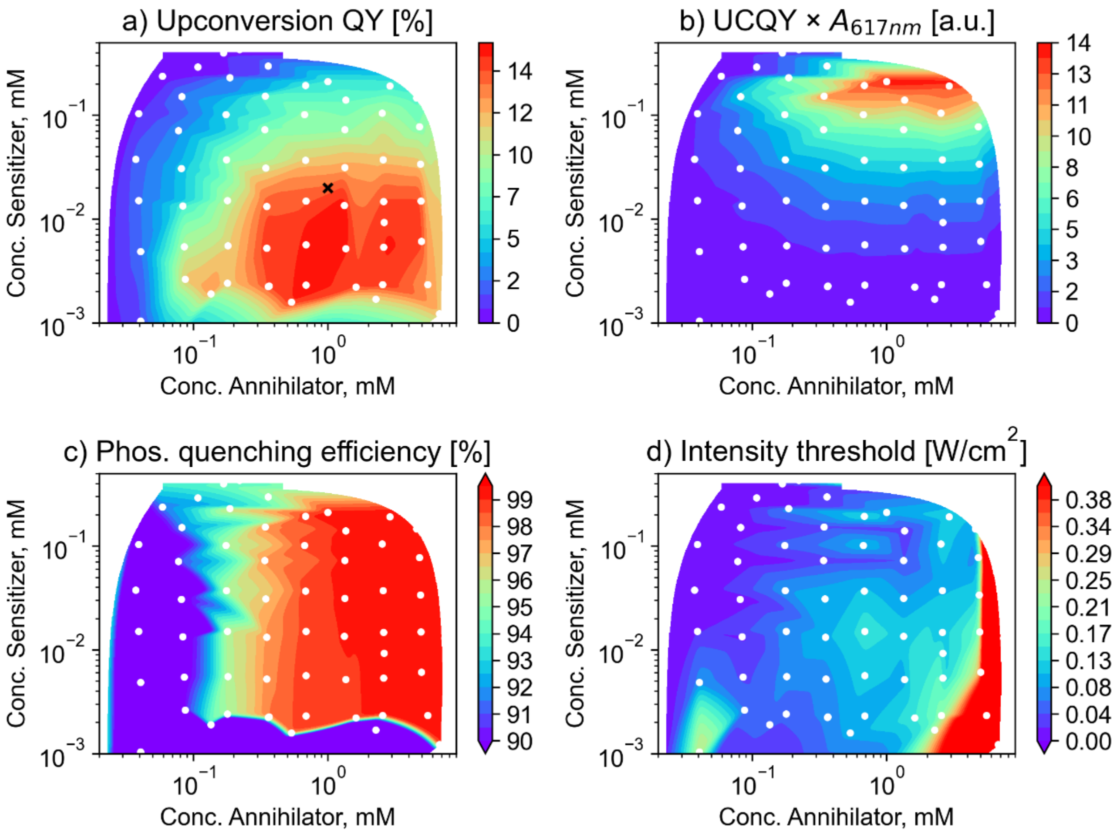

Figure S11. Concentration mapping of main TTAUC parameters in Pt(TPBP) sensitizer and Anc-mono annihilator system. (a) Upconversion quantum yield. Black cross indicates position at 20 µM sensitizer and 1 mM annihilator concentration. (b) Potential brightness corresponding to upconverted emission output per incident photons at peak sensitizer absorbance of 617 nm. (c) Phosphorescence quenching efficiency calculated with 70% intrinsic phosphorescence efficiency of Pt(TPBP). (d) Excitation intensity threshold power for 633 nm excitation determined at half upconversion efficiency. Concentrations were estimated from absorbance measurements (Figure S11c).

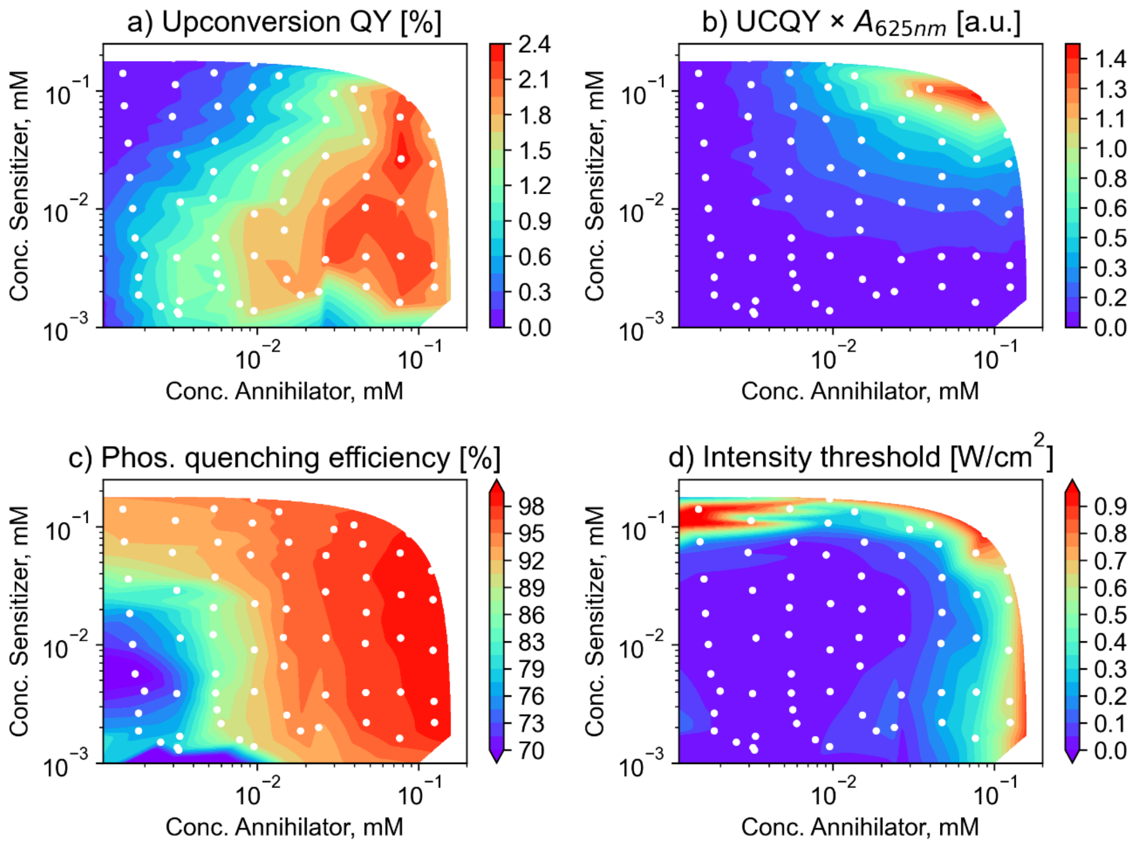

Figure S12. Concentration mapping of main TTAUC parameters in Pd(TPBP) sensitizer and Anc-hex annihilator system. (a) Upconversion quantum yield. (b) Potential brightness corresponding to upconverted emission output per incident photons at peak sensitizer absorbance of 625 nm. (c) Phosphorescence quenching efficiency calculated with 9% intrinsic phosphorescence efficiency of Pd(TPBP). (d) Excitation intensity threshold power for 633 nm excitation determined at half upconversion efficiency. Low sensitizer concentration values (< 0.01 mM) were distorted by scattering of Anc-hex aggregates above 0.02 mM annihilator concentration. Concentrations were estimated from absorbance measurements (Figure S11b).

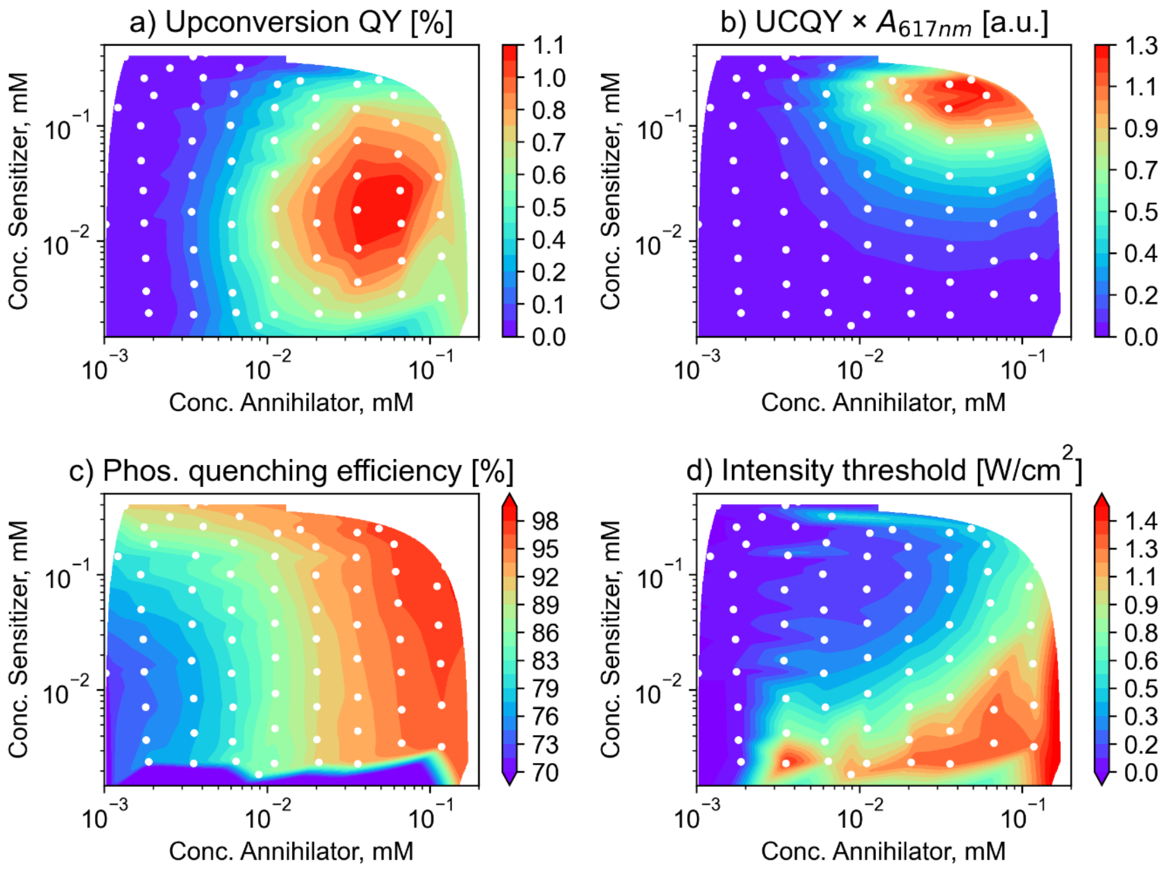

Figure S13. Concentration mapping of main TTAUC parameters in Pt(TPBP) sensitizer and Anc-hex annihilator system. (a) Upconversion quantum yield. (b) Potential brightness corresponding to upconverted emission output per incident photons at peak sensitizer absorbance of 617 nm. (c) Phosphorescence quenching efficiency calculated with 70% intrinsic phosphorescence efficiency of Pt(TPBP). (d) Excitation intensity threshold power for 633 nm excitation determined at half upconversion efficiency. Low sensitizer concentration values (< 0.01 mM) were distorted by scattering of Anc-hex aggregates above 0.02 mM annihilator concentration. Concentrations were estimated from absorbance measurements (Figure S11d).

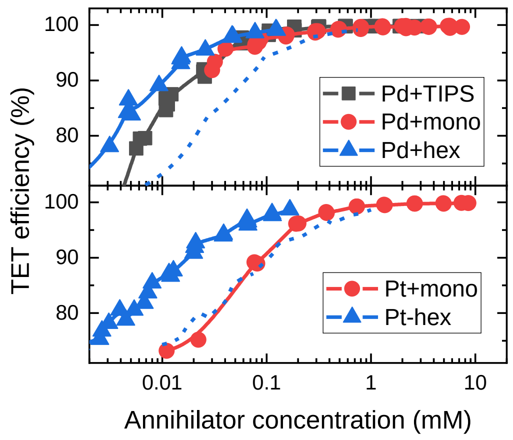

Figure S14. Triplet energy transfer (TET) efficiency as a function of annihilator concentration. TET efficiency plotted for different combinations of two sensitizers (a) Pd(TPBP) and (b) Pt(TPBP), and three different annihilators: TIPS-Anthracene, Anc-mono and Anc-hex. Dashed blue line represents TET efficiency with Anc-hex annihilator at effective concentration of 6 anthracene units per molecules. For all of the TET efficiency plots sensitizer concentration range was set to 3-6 µM.

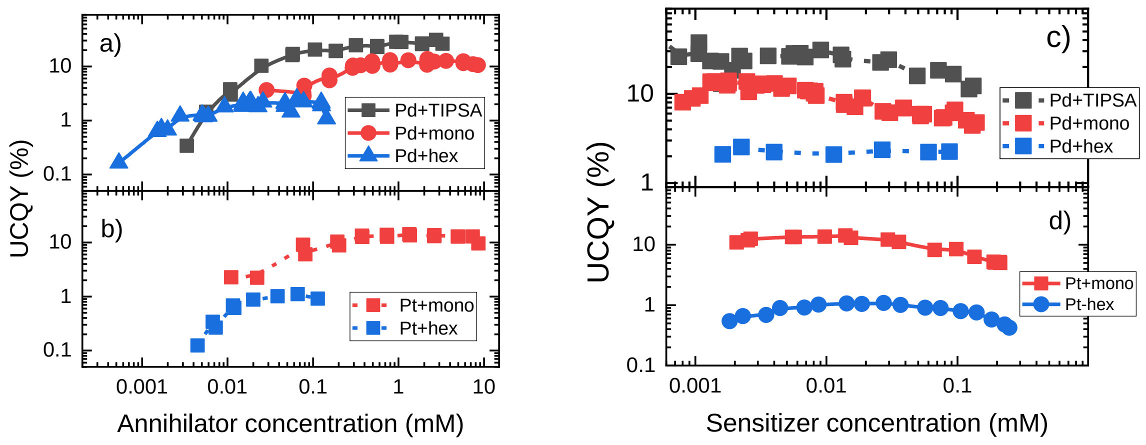

Figure S15. Upconversion quantum yield (UCQY) plots as a function of (a, b) annihilator concentration and (c, d) sensitizer concentration. Annihilators TIPS-antracene, Anc-mono and Anc-hex were tested with Pd(TPBP) sensitizer (a, c) and annihilators Anc-mono and Anc-hex were tested with Pt(TPBP) sensitizer (b, d). Displayed UCQY values were recorded at a narrow concentration range of sensitizer concentrations 1-10 µM (a, b) or annihilator concentrations 0.5-2 mM for Anc-mono and 0.06-0.1 mM for Anc-hex (c, d) reflecting cross-section with the highest attained UCQY values.

6. Theoretical calculations of triplet energies

All molecular structures were optimized using density functional theory (DFT) with Gaussian 16, revision C.01. To reduce computational cost and time, the bulky triisopropylsilyl (TIPS) groups present in some compounds were replaced with trimethylsilyl (TMS) groups for the calculations. This substitution is considered acceptable because the electronic effects of TMS and TIPS groups are largely similar, and replacing TIPS with TMS does not significantly alter the electronic properties or frontier molecular orbitals of the molecules.

B3LYP/6-31G(d) level was used for all three compounds as it gives reasonable compromise between computational time and quality of results. For Anc-mono, TMS-Anc and Anc-hex, the B3LYP/6-31G(d) level of theory was employed, as it provides a reasonable compromise between computational efficiency and the quality of the results.[10] Figure S18 presents calculated singlet and triplet lowest state energies (S_1 and T_1, respectively) in relation to experimentally obtained values. Experimental S_1 energies were obtained from absorption edge. Experimental T_1 of 1.37 eV for TIPS-Anc of was reported.[11] B3LYP/6-31G(d) level DFT calculations underestimated both singlet and triplet states relative to experimental values (Figure S18). Therefore, for comparative purposes, only the relative energy differences between the molecules should be considered.

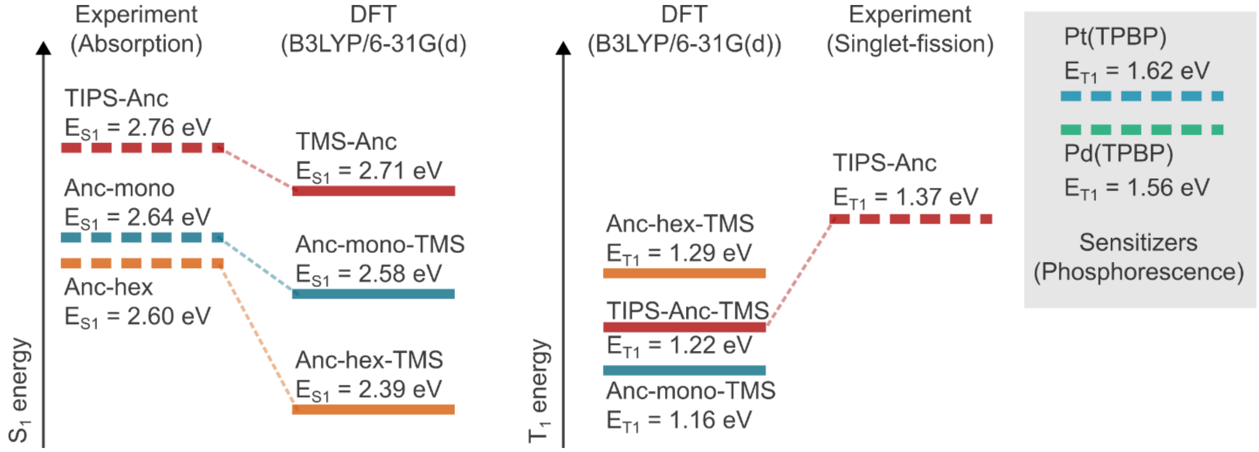

Figure S16. Energy diagram of DFT calculated and experimentally obtained lowest singlet (S_1) and triplet (T_1) energy states for TIPS-Anc, Anc-mono and Anc-hex. Experimental triplet energies for photosensitizers used in this work are indicated in a grey box.

7. Aggregation of hexamer annihilator

At higher concentrations hexamers exhibited aggregate formation, as evidenced by a new emission band at 540 nm and strong light scattering (Figure S17). This aggregation also led to a gradual increase in I_{th} with increasing annihilator concentration in the **Anc-hex:Pd(TPBP)** system, coinciding with the appearance of new absorption and emission bands (Figure S18). These factors could have caused erroneous determination of maximum ϕ_{UC}.

The lower maximum ϕ_{UC} for **Anc-hex** with **Pt(TPBP)** sensitizer (ϕ_{UC} = 1.1%) compared to **Pd(TPBP)** sensitizer (ϕ_{UC} = 2.7%) was likely due to combination of higher aggregate content and the increased excitation threshold. Excimer emission band was more pronounced in **Pt(TPBP)** samples (Figure S17a) compared to **Pd(TPBP)** samples (Figure S18f) at similar **Anc-hex** concentrations, which could be influenced by different external environmental factors such as different temperature and storage time of prepared solution. Furthermore, excitation threshold for **Anc-hex** with **Pt(TPBP)** exceeded 1 W/cm² (Figure S13), which was close to the maximum excitation power densities achievable with our system. In comparison, the excitation threshold for **Anc-hex** with **Pd(TPBP)** was almost an order of magnitude lower (Figure S12), largely due to the significant increase in the absorption coefficient at the laser excitation wavelength.

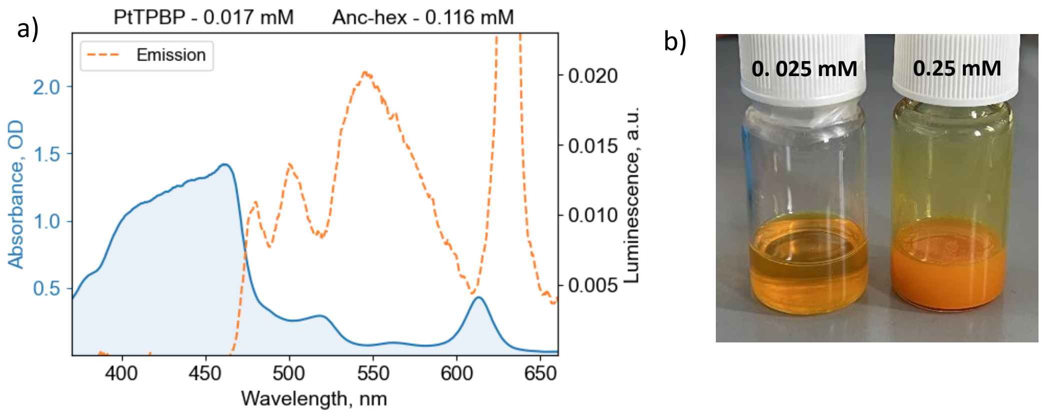

Figure S17. Formation of Anc-hex aggregates. (a) absorbance and upconverted emission spectrum at specified Pt(TPBP) sensitizer and Anc-hex annihilator concentrations. (b) pictures of concentrated Anc-hex solutions showing the effects of aggregate scattering.

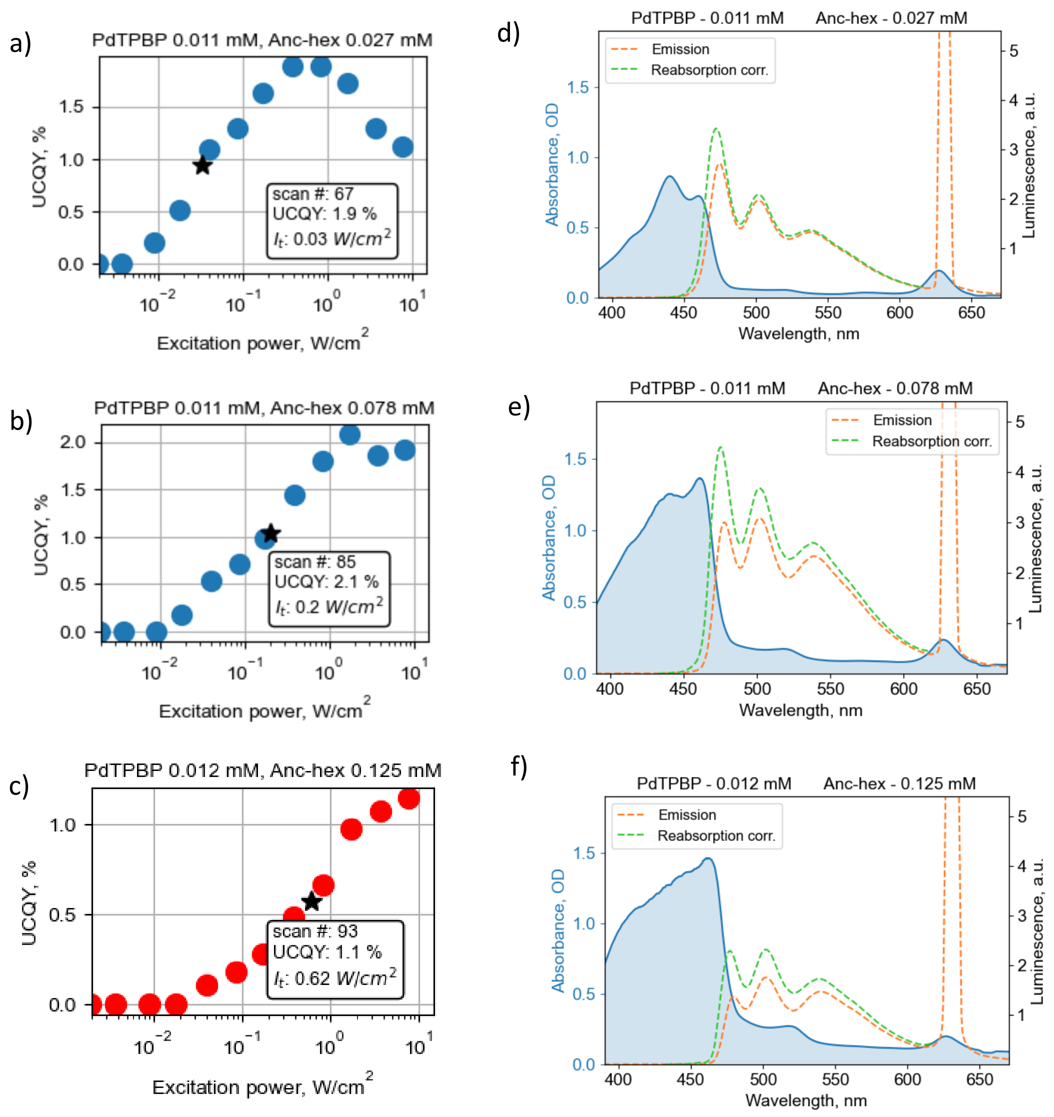

Figure S18. Excitation threshold increase due to Anc-hex aggregate formation. (a-c) Upconversion quantum yield versus excitation power at increasing Anc-hex concentration. (c-d) Corresponding absorbance and photoluminescence spectra. Aggregate formation is associated with formation of new absorption and emission bands in 500-550 nm region.

8. Determination of Anc-Mono and Anc-Hex spin-statistical factors

The spin-statistical factors of Anc-Mono and Anc-Hex were determined using datapoints of PdTPBP:Anc-Mono (3 µM and 2 mM respectively) and PdTPBP:Anc-Hex (11 µM and 0.027 mM respectively) UC solutions in toluene. We also included calculation of reference sample PdTPBP:TIPS-Anc (3 µM and 2 mM respectively). In the selected sensitizer concentration range (3-11 µM) the detrimental effects to the ϕ_{UC} due to sensitizer aggregation, sensitizer TTA or FRET-induced quenching are minimal (Figure S19). Similarly, FRET-induced quenching of ϕ_{FL} is negligible. The spin-statistical factors were calculated as follows:

$$f = \frac{2\phi_{UC}}{\phi_{ISC}\phi_{TET}\phi_{TTA}\phi_{FL}} \tag{1}$$

ϕ_{ISC} of PdTPBP sensitizer was reported to be 97% in the previous work.[12] We assume that ϕ_{TTA} approaches unity as the maximum ϕ_{UC} values were measured at excitation densities more than order of magnitude higher than the excitation thresholds listed in Table S1. For the calculation of spin-statistical factor for Anc-hex, the annihilator concentration prior to aggregate formation (Figure S20a). Given that the annihilator concentration is comparable to oxygen concentration (5 µM), the ϕ_{TET} may be overestimated, which would result in underestimation of spin-statistical factor in the case of Anc-hex in Table S1.

Table S1. Calculated values of the photophysical TTA-UC parameters. ϕ_{TET}, I_{th} and ϕ_{UC} were estimated by automated screening experiments. ϕ_{FL} was estimated separately using an integrating sphere method.

**Sensitizer**	**Annihilator**	$\boldsymbol{\phi_{FL}}$ (%)	$\boldsymbol{\phi_{TET}}$(%)	$\boldsymbol{I_{th}}$ (mW/cm^2)	$\boldsymbol{\phi_{UC}}$(%)	$\boldsymbol{f}$(%)
PdTPBP (6 µM)	TIPS-Anc (1mM)	98	96.6	80	28.5 (at 5 W/cm^2)	59.9
PdTPBP (3 µM)	Anc-Mono (2mM)	95	98.5	120	13 (at 5 W/cm^2)	29.9
PdTPBP (11 µM)	Anc-Hex (0.03 mM)	84	94.0	30	1.9 (at 0.5 W/cm^2)	5.0

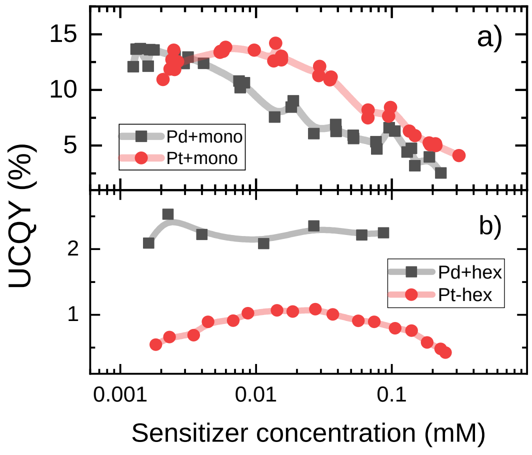

Figure S19. Upconversion quantum yield (UCQY) plots as a function of sensitizer concentration. Two different annihilator (a) Anc-mono and (b) Anc-hex were tested with Pd(TPBP) and Pt(TPBP) sensitizers. Displayed UCQY values were recorded at a narrow annihilator concentration range of (a) 0.5-2 mM for Anc-mono and (b) 0.07-0.1 mM for Anc-hex to represent maximum UCQY values.

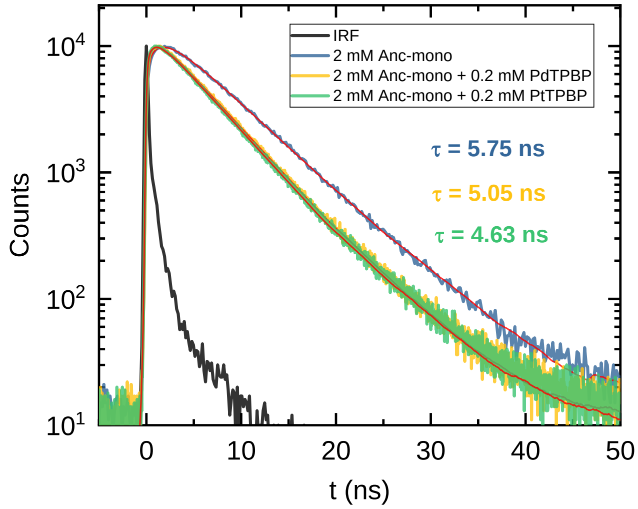

Figure S20. Annihilator fluorescence transients to show Forster resonant energy transfer (FRET) induced losses. High sensitizer concentration of 0.2 mM resulted in noticeable FRET due to overlap of annihilator emission and sensitizer absorbance signal. Differences for Pt(TPBP) and Pd(TPBP) sensitizers were negligible. Samples were excited with 420 nm laser, emission transients were recorded at 490 nm emission. Fluorescence transients were measured under ambient oxygen conditions to minimize effects of triplets.

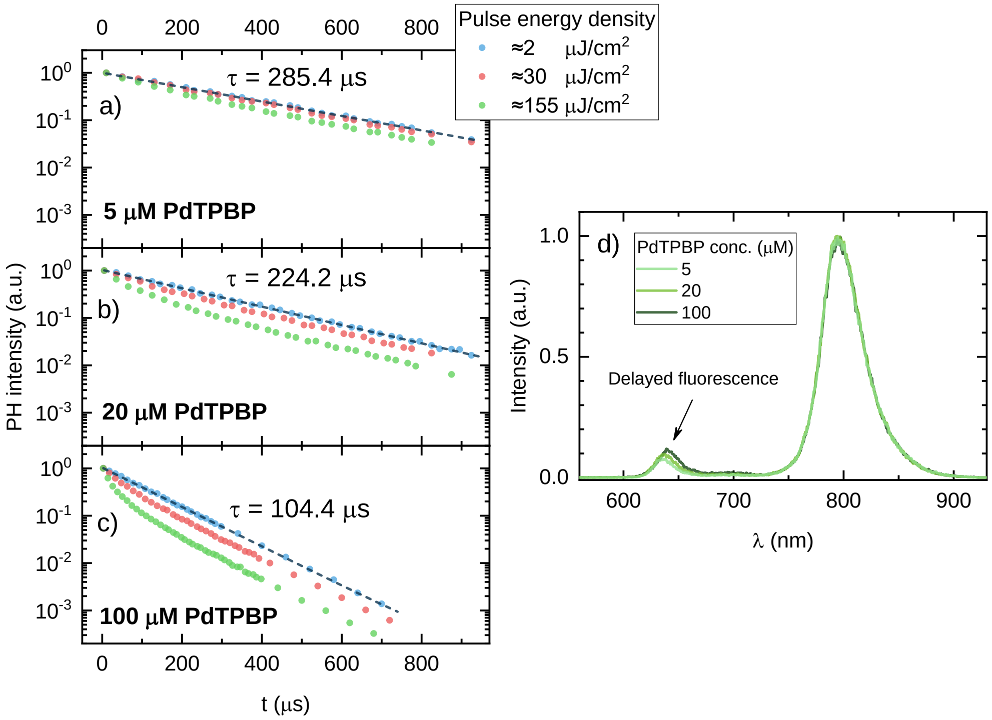

Figure S21. (a-c) Phosphorescence emission transients of 5 µM, 20 µM and 100 µM PdTPBP solutions in toluene (respectively) at different excitation pulse energy densities. Dotted lines represent exponential fits. The samples were excited using a 640 nm pulsed laser (1 kHz repetition rate). The emission spectra were integrated in the range of 770-830 nm. (d) proof of PdTPBP delayed emission at ≈10 µs after the excitation pulse occurring due to the sensitizer triplet-triplet annihilation (STTA). The spectra were measured using 155 µJ/cm^2 pulse energy density.

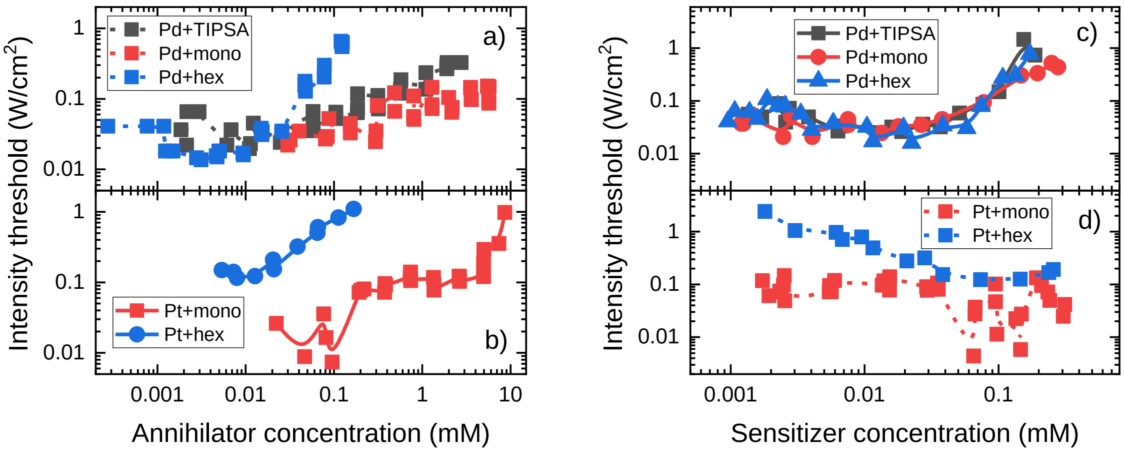

Figure S22. Intensity threshold plots as a function of (a, b) annihilator concentration and (c, d) sensitizer concentration. Annihilators TIPS-antracene, Anc-mono and Anc-hex were tested with Pd(TPBP) sensitizer (a, c) and annihilators Anc-mono and Anc-hex were tested with Pt(TPBP) sensitizer (b, d). Displayed thresholds were recorded at a narrow concentration range of sensitizer concentrations (a, b) or annihilator concentrations (c, d) reflecting the lowest attained excitation threshold values.

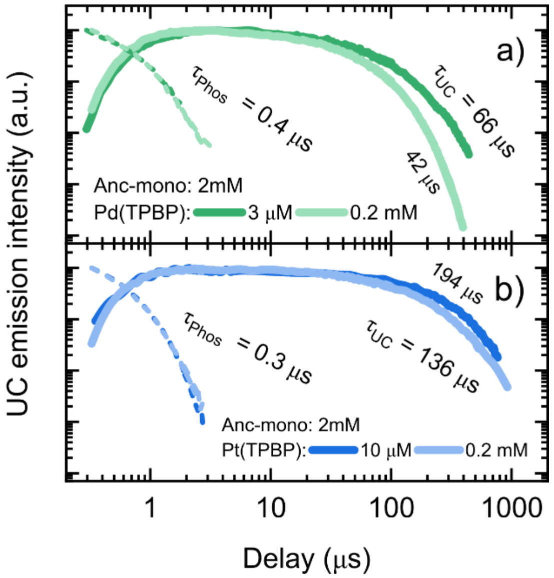

Figure S23. Upconverted emission transients integrated in 480-520 nm emission region for same 2mM Anc-mono annihilator concentration and (a) 3 µM and 0.2 mM Pd(TPBP), (b) 10 µM and 0.2 mM Pt(TPBP) sensitizer concentrations. Sensitizer phosphorescence transients integrated in 750-850 nm emission region are added for reference and indicated by dashed lines. Fitted single exponential decay lifetimes for phosphorescence and upconverted emission are indicated.

9. Synthesis of Anc-mono and Anc-hex

Materials and General Methods

Reagents (Acros, Aldrich, Fischer, BLD, Angene, Ambeed, and TCI) were purchased as reagent grade and used without further purification.

Solvents for extraction or column chromatography were used analytical grade.

Dry solvents (THF, CH_2Cl_2, diethyl ether, and toluene) for reactions were purified by a solvent drying system from MBraun under nitrogen atmosphere (H_2O content < 10 ppm as determined by Karl-Fischer titration). All other solvents were purchased in p.a. quality.

Reactions in the absence of air and moisture were performed in oven-dried glassware under Ar atmosphere.

Flash column chromatography (FC) was performed using Biotage® Selekt apparatus at 25 °C with a head pressure of 0.0–30 bar and Flow Rate (50-250 mL/min). SiO_2 (60 Å, 230–400 mesh, particle size 0.040–0.063 mm, Fluka). The used solvent compositions are reported in synthetic procedures.

Analytical thin layer chromatography (TLC) was performed on aluminum sheets coated with silica gel 60 F254 (Merck, Macherey-Nagel). Visualization was achieved using UV light (254 or 365 nm).

Evaporation in vacuo was performed at 25–60 °C and 800–10 mbar.

Reported yields refer to spectroscopically and chromatographically pure compounds that were dried under high vacuum (0.5–0.1 mbar) before analytical characterization.

^{1}H and ^{13}C nuclear magnetic resonance (NMR) spectra were recorded on Bruker 400 (Avance III HD), Bruker DRX 500, Varian-Agilent 500 and Varian-Agilent 600 spectrometers at 400 MHz, 500 MHz or 600 MHz (^{1}H) and 75 MHz, 126 MHz or 150 MHz (^{13}C), respectively. Temperatures of measurements are indicated in the procedures and on the spectra. Chemical shifts δ are reported in ppm downfield from tetramethylsilane using the residual deuterated solvent signals as an internal reference ($CDCl_3$: δH = 7.26 ppm, δC = 77.0 ppm). For ^{1}H NMR, coupling constants J are given in Hz and the resonance multiplicity is described as s (singlet), d (doublet), t (triplet), q (quartet), m (multiplet). All spectra were recorded at 298 K.

Mass spectrometry (MS) was performed by the Laboratory for Analysis of Bioactive Compounds at the Institute of Organic Chemistry PAS on a AutoSpec Premier spectrometer (EI), on a 4000 Q-TRAP spectrometer (ESI) and (ACPI), or in the Department of Chemistry and Polymer Technology, Faculty of Chemistry, Warsaw University of Technology at Bruker UltrafleXtreme spectrometer (MALDI). For MALDI measurements, the matrix was trans-2-[3-(4-tert-Butylphenyl)-2-methyl-2-propenylidene]malononitrile (DCTB) with addition of potassium trifluoroacetate.

Melting Points All melting points for crystalline products were measured with automated melting point apparatus EZ-MELT and were given without correction.

Synthetic protocols

10-hydroxy-10-((triisopropylsilyl)ethynyl)anthracen-9(10H)-one (S1)

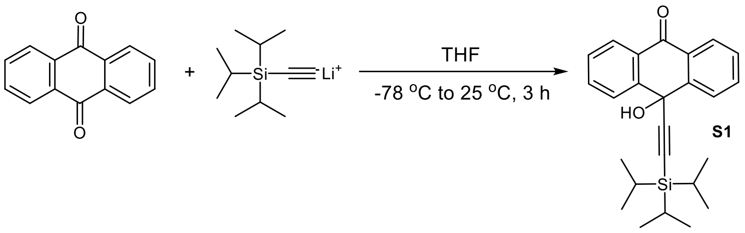

n-BuLi (2.5 M in hexanes, 4.0 mL, 10.00 mmol) was added dropwise to a solution of (triisopropylsilyl)acetylene (2.16 mL, 10.10 mmol) in THF (20 mL) at –79 °C. The solution was stirred for 30 min before being transferred slowly via cannula into a solution of 9,10-anthraquinone (2.0 g, 9.61 mmol) in THF (50 mL) at 0 °C. After 3 h at 25 °C, the reaction was quenched at 0 °C with satd. aq. NH_4Cl (30 mL). Resulting mixture was extracted with EtOAc (3 x 20 mL), washed with brine (20 mL), dried over Na_2SO_4, and the solvent removed in vacuo. The residue was recrystallized from hexane/CH_2Cl_2 to give **S1** as a white powder in 89% yield (3.34 g, 8.55 mmol).

mp: 155 °C

^{1}H NMR: (500 MHz, $CDCl_3$) δ 8.22 (d, *J* = 7.8 Hz, 2H; H-C(Anthr)), 8.12 (d, *J* = 7.9 Hz, 2H; H-C(Anthr)), 7.71 (t, *J* = 7.4 Hz, 2H; H-C(Anthr)), 7.52 (t, *J* = 7.5 Hz, 2H; H-C(Anthr)), 3.04 (s, 1H; OH), 1.03 ppm (s, 21H; TIPS)

^{13}C NMR: (126 MHz, $CDCl_3$) δ 182.98, 143.63, 133.99, 129.25, 129.09, 128.20, 127.17, 108.78, 88.35, 66.48, 18.54, 11.14 ppm

HR-AP-MS m/z: 389.1938 ($[M-H]^-$, calcd for $C_{25}H_{29}O_2Si^-$: 389.1937)

((9,10-dimethoxy-10-((triisopropylsilyl)ethynyl)-9,10-dihydroanthracen-9-yl)ethynyl)trimethylsilane (S2)

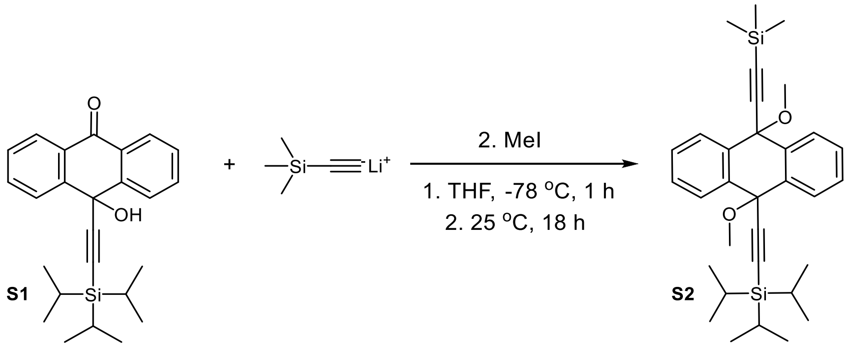

n-BuLi (2.5 M in hexanes, 8.96 mL, 22.40 mmol) was added dropwise to a solution of trimethylsilylacetylene (3.10 mL, 22.40 mmol) in THF (35 mL) at –79 °C. The solution was stirred for 20 min before being transferred slowly via cannula into a solution of **S1** (2.5 g, 6.40 mmol) in THF (60 mL) at 0 °C. After 4 h at 25 °C, reaction mixture was cooled to –20 °C and MeI (3.19 mL, 51.20 mmol, 8 equiv.) was added dropwise. After 18 h stirring at 25 °C, the reaction was quenched at 0 °C with satd. aq. NH_4Cl (40 mL). Resulting mixture was extracted with EtOAc (3 x 25 mL), washed with brine (30 mL), dried with Na_2SO_4, and the solvent removed in vacuo. The residue was recrystallized from CH_2Cl_2/MeOH to give **S2** as a white powder in 84% yield (2.78 g, 5.70 mmol).

mp: 95 °C

^{1}H NMR: (600 MHz, $CDCl_3$) δ 8.02 – 7.97 (m, 2H; H-C(Anthr)), 7.94 – 7.89 (m, 2H; H-C(Anthr)), 7.50 – 7.44 (m, 4H; H-C(Anthr)), 2.81 (s, 3H; OMe), 2.75 (s, 3H; OMe), 1.02 (s, 21H; TIPS), 0.11 ppm (s, 9H; TMS)

^{13}C NMR: (151 MHz, $CDCl_3$) δ 135.85, 135.63, 128.96, 128.69, 128.21, 127.93, 108.63, 107.11, 90.58, 88.50, 77.19, 76.98, 76.77, 72.86, 72.31, 51.14, 50.98, 18.53, 11.16, - 0.25 ppm

HR-ESI-MS: m/z: 539.2779 ([M+Na]$^+$, calcd for $C_{32}H_{44}O_2NaSi_2^+$: 539.2778)

((10-ethynyl-9,10-dimethoxy-9,10-dihydroanthracen-9-yl)ethynyl)triisopropylsilane (S3)

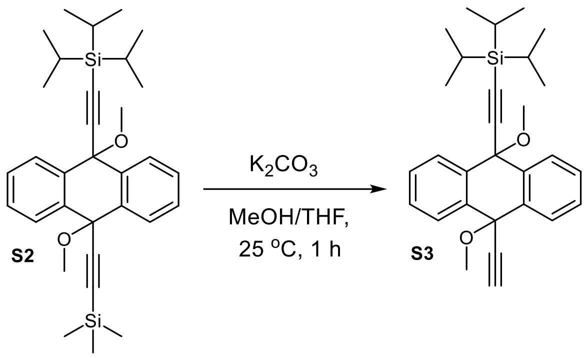

K_2CO_3 (1.00 g, 7.25 mmol) was added to solution of **S2** (2.5 g, 4.84 mmol) in THF-MeOH mixture (60 mL, 6:4, v/v) at 25 °C and stirred for 1.5 h. Then, CH_2Cl_2 (30 mL) was added and the resulting mixture was passed through an SiO_2 plug and eluted with CH_2Cl_2 (30 mL). Solvents were evaporated in vacuo leading to crude product. Obtained residue was dissolved in CH_2Cl_2 (25 mL) and precipitated by the addition of cold MeOH (100 mL). The solid was washed with cold MeOH (2 × 10 mL) to give **S3** as an off-white solid in 94% yield (2.02 g, 4.55 mmol).

mp: 114 °C

^{1}H NMR: (600 MHz, $CDCl_3$) δ 8.04 – 8.00 (m, 2H; H-C(Anthr)), 7.95 – 7.90 (m, 2H; H-C(Anthr)), 7.51 – 7.46 (m, 4H; H-C(Anthr)), 2.85 (s, 3H; OMe), 2.76 (s, 3H; OMe), 2.66 (s, 1H; H-C≡C), 1.04 ppm (s, 21H; TIPS)

^{13}C NMR: (151 MHz, $CDCl_3$) δ 135.88, 135.18, 129.15, 128.86, 128.39, 127.78, 108.16, 89.15, 86.22, 73.65, 72.99, 71.63, 51.19, 50.97, 18.54, 11.17 ppm

HR-ESI-MS: m/z: 467.2376 ([M+Na]$^+$, calcd for $C_{29}H_{36}O_2NaSi^+$: 467.2382)

[2-(10-{2-[4''-(2-{9,10-Dimethoxy-10-[2-(triisopropylsilyl)ethynyl]-9,10-dihydro-9-anthryl}ethynyl)-3',4',5',6'-tetrakis[p-(2-{9,10-dimethoxy-10-[2-(triisopropylsilyl)ethynyl]-9,10-dihydro-9-anthryl}ethynyl)phenyl]-o-terphenyl-4-yl]ethynyl}-9,10-dimethoxy-9,10-dihydro-9-anthryl)ethynyl]triisopropylsilane (S5)

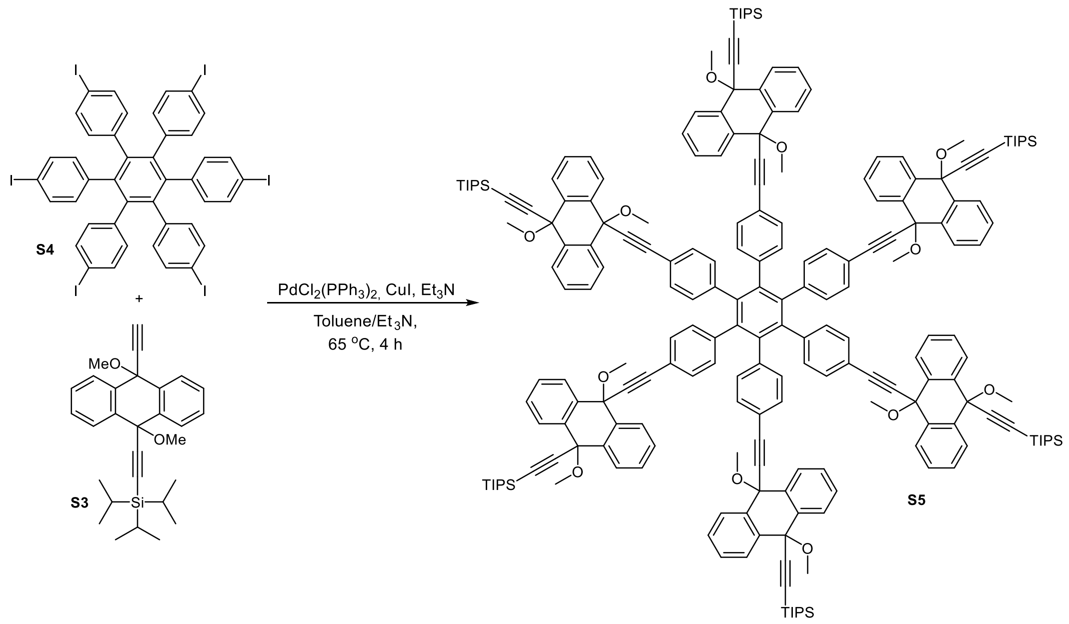

Anthracene derivative **S3** (496.3 mg, 1.12 mmol) was added to a degassed solution of iodoarene **S4**[13](200 mg, 0.155 mmol) in a toluene-Et_3N mixture (18 mL, 7:2, v/v). Then, $[Pd(PPh_3)_2Cl_2]$ (43.5 mg, 0.062 mmol) and CuI (14.7 mg, 0.078 mmol) were added. Flushing with Ar was continued for further 5 min The reaction was stirred at 65 °C under Ar for 4 h. After cooling to 25 °C, CH_2Cl_2 (20 mL) was added. The reaction mixture was passed through SiO_2 pad and eluted with CH_2Cl_2 (25 mL). Solvents were evaporated in vacuo. Solid residue was purified by recrystallization from hexane to remove homocoupling byproduct. Then solid was recrystallized from acetone/MeOH to give **S4** as a pale yellow solid in 81% yield (400.6 mg, 0.13 mmol).

mp: 251 °C (decomp)

^{1}H NMR: (500 MHz, $CDCl_3$) δ 7.98 – 7.88 (m, 24H; H-C(Anthr)), 7.47 – 7.39 (m, 24H; H-C(Anthr)), 6.86 (d, J = 8.4 Hz, 12H; H-C(Ar)), 6.54 (d, J = 8.4 Hz, 12H; H-C(Ar)), 2.75 (s, 18H, OMe), 2.73 (s, 18H, OMe), 1.01 ppm (s, 126H, TIPS)

^{13}C NMR: (126 MHz, $CDCl_3$) δ 139.94, 139.71, 135.87, 135.50, 130.75, 130.60, 129.07, 128.74, 128.25, 128.13, 119.74, 109.07, 91.37, 88.20, 85.77, 72.61, 72.36, 51.17, 50.97, 18.53, 11.16 ppm

MALDI-MS: m/z: 3228.497 ($[M+K]^+$, calcd for $C_{216}H_{234}KO_{12}Si_6^+$: 3228.601)

Triisopropyl[2-(10-{2-[4''-(2-{10-[2-(triisopropylsilyl)ethynyl]-9-anthryl}ethynyl)-3',4',5',6'-tetrakis[p-(2-{10-[2-(triisopropylsilyl)ethynyl]-9-anthryl}ethynyl)phenyl]-o-terphenyl-4-yl]ethynyl}-9-anthryl)ethynyl]silane (S6 or Anc-hex)

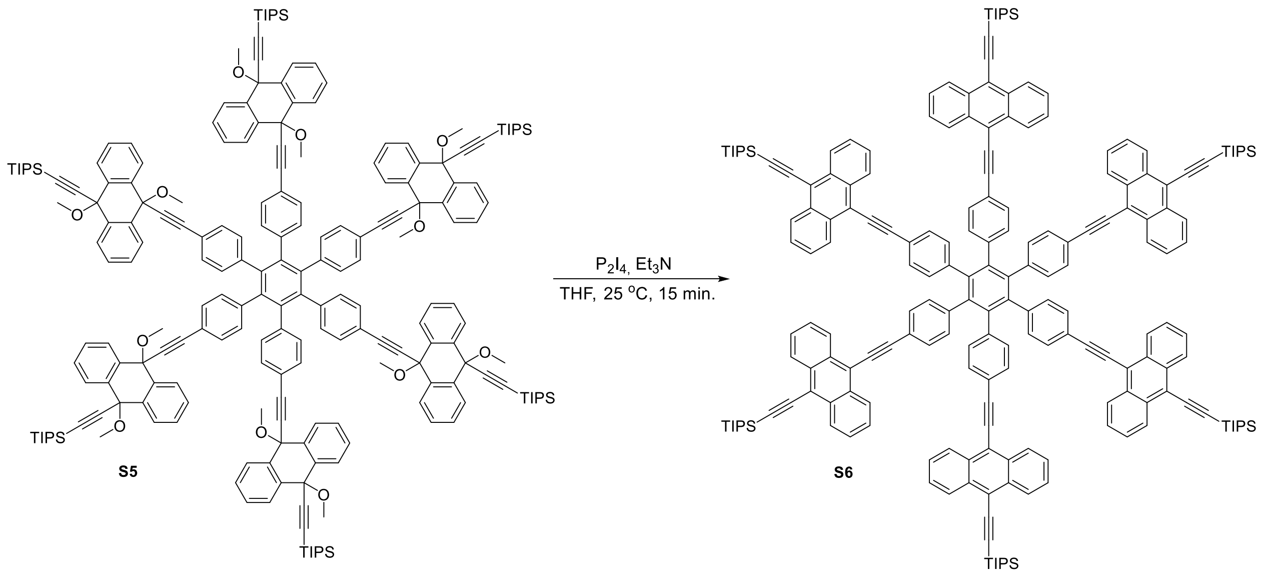

P_2I_4 (219 mg, 0.38 mmol) was added in one portion to the solution of **S4** (350 mg, 0.11 mmol) in THF (25 mL) deoxygenated with Ar. The solution was stirred for 15 min at 25 °C before Et_3N (122 µL, 0.88 mmol) was added. Then, the solvent was evaporated in vacuo to obtain orange solid. The solid was suspended in MeOH (20 mL) sonicated, filtered and washed with methanol (15 mL) and cold acetone (10 mL) to give **Anc-hex (S6)** as an orange crystalline solid in 92% yield (285 mg, 0.10 mmol).

mp: > 300 °C (decomp)

^{1}H NMR: (600 MHz, $CDCl_3$) δ 8.59 – 8.54 (m, 24H; H-C(Anthr)), 7.51 – 7.45 (m, 36H; H-C(Anthr, Ar)), 7.09 (d, *J* = 8.4 Hz, 12H; H-C(Ar)), 1.23 ppm (s, 126H; TIPS)

^{13}C NMR: (1510 MHz, $CDCl_3$) δ 140.46, 140.27, 132.40, 131.87, 131.58, 130.77, 127.14, 126.77, 126.65, 121.06, 118.54, 118.34, 104.77, 103.35, 102.57, 86.94, 18.84, 11.47 ppm

MALDI-MS: m/z: 2818.357 ($[M+H]^+$, calcd for $C_{204}H_{199}Si_6^+$: 2818.425)

5-(Phenylethynyl)-12-((triisopropylsilyl)ethynyl)-5,12-dihydrotetracene-5,12-diol (S5)

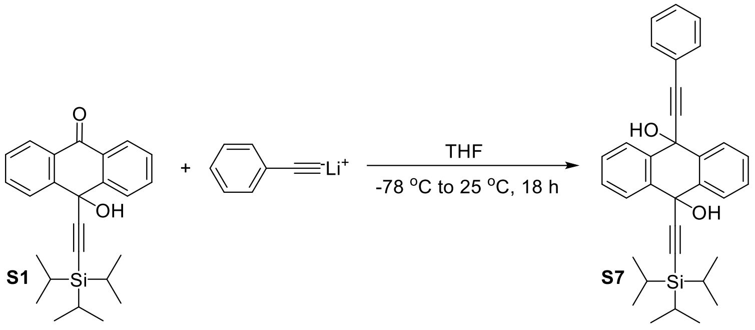

n-BuLi (2.5 M in hexanes, 4.61 mL, 11.52 mmol) was added dropwise to a solution of phenylacetylene (1.47 µL, 11.52 mmol) in THF (40 mL) at –79 °C. The solution stirred for 40 min at –79 °C before **S1** (1.5 g, 3.84 mmol) was added. After 18 h, the reaction was quenched at 0 °C with satd. aq. NH_4Cl (30 mL). Resulting mixture was extracted with EtOAc (2 x 20 mL), washed with brine (15 mL), dried with Na_2SO_4, and the solvent removed in vacuo. The residue was purified by column chromatography (SiO_2, Hexane /AcOEt (9:1 -> 8:2)) to afford product **S7** as a pale yellow oil in 82% yield (1.55 g, 3.14 mmol).

^{1}H NMR: (500 MHz, $CDCl_3$) δ 8.18 – 8.10 (m, 4H; Ar), 7.51 – 7.45 (m, 4H; H-C(Anthr)), 7.43 – 7.38 (m, 2H; H-C(Anthr)), 7.29 – 7.24 (m, 3H; H-C(Anthr, Ar)), 3.11 (s, 1H; OH), 2.98 (s, 1H; OH), 1.13 – 1.03 ppm (m, 21H; TIPS)

^{13}C NMR: (126 MHz, $CDCl_3$) δ 138.31, 137.55, 131.70, 129.18, 129.00, 128.69, 128.19, 126.95, 126.68, 122.04, 109.27, 91.51, 89.18, 86.57, 68.62, 68.10, 18.60, 11.20 ppm

HR-APCI-MS: m/z: 475.2459 ([M-H_2O]$^+$, calcd for $C_{33}H_{35}OSi^+$: 475.2457)

Triisopropyl((12-(phenylethynyl)tetracen-5-yl)ethynyl)silane (Anc-mono or S8)

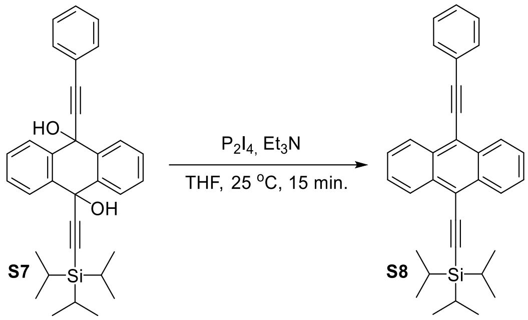

P_2I_4 (127 mg, 2.23 mmol) was added in one portion to a solution of **S7** (1 g, 2.03 mmol) in THF (50 mL) deoxygenated with Ar. The solution was allowed to stir for 15 min at 25 °C before Et_3N (622 µL, 4.47 mmol) was added. Then, solvents were evaporated in vacuo. The residue was purified by recrystallization from acetone/MeOH, filtered and solids washed with methanol (15 mL) to give **Anc-mono** (**S8)** as a yellow crystalline solid in 93% yield (866 mg, 1.89 mmol).

mp: 85°C

^{1}H NMR: (500 MHz, $CDCl_3$) δ 8.73 – 8.62 (m, 4H; Ar), 7.80 – 7.75 (m, 2H; H-C(Anthr)), 7.63 (dd, *J* = 6.8, 3.1 Hz, 4H; H-C(Anthr)), 7.48 – 7.40 (m, 3H; H-C(Anthr, Ar)), 1.28 ppm (s, 21H; TIPS)

^{13}C NMR: (126 MHz, $CDCl_3$) δ 132.51, 132.01, 131.69, 128.69, 128.56, 127.29, 127.20, 126.88, 126.73, 123.41, 118.71, 118.46, 104.90, 103.36, 102.33, 86.45, 18.89, 11.52 ppm

HR-APCI-MS: m/z: 459.2507 ($[M+H]^+$, calcd for $C_{33}H_{35}Si^+$: 459.2508)

^{1}H and ^{13}C NMR spectra

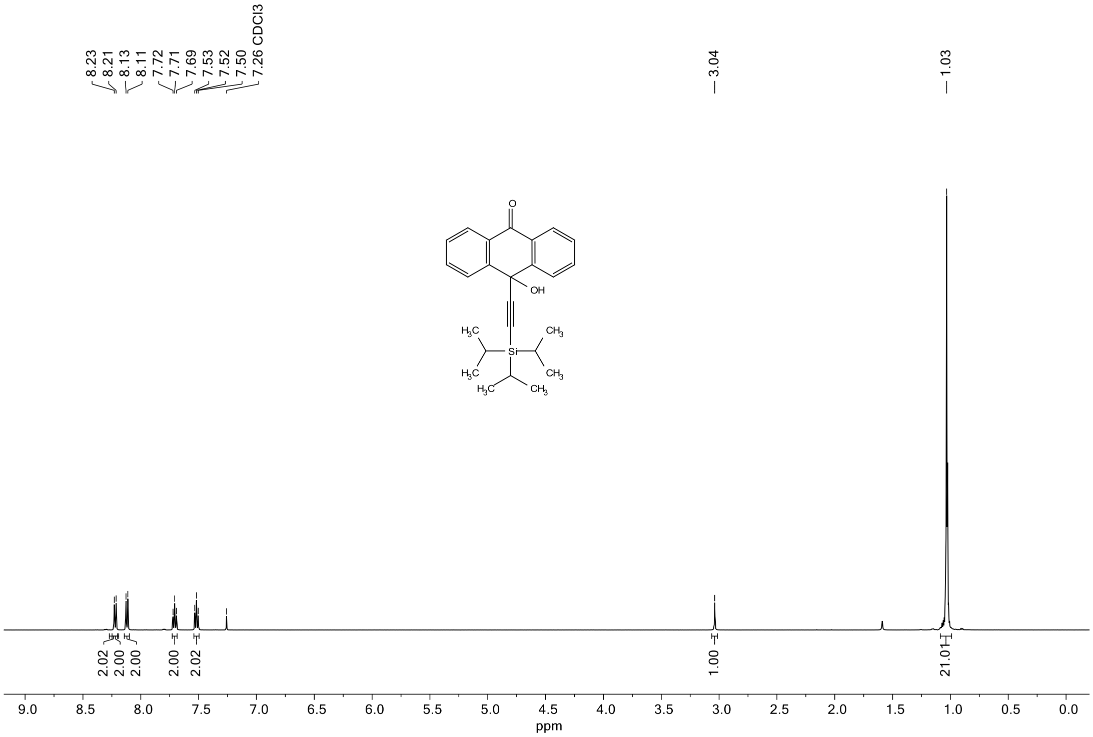

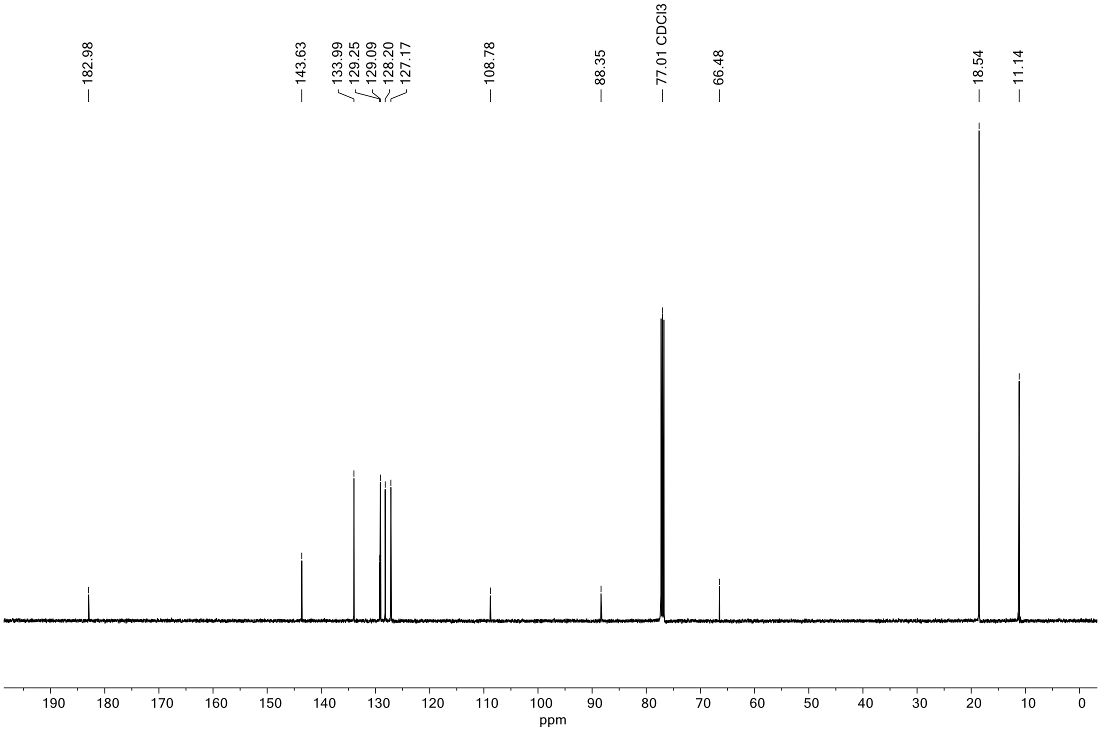

Figure S24. ^{1}H NMR (top) and ^{13}C NMR (bottom) spectra of **S1** in $CDCl_3$.

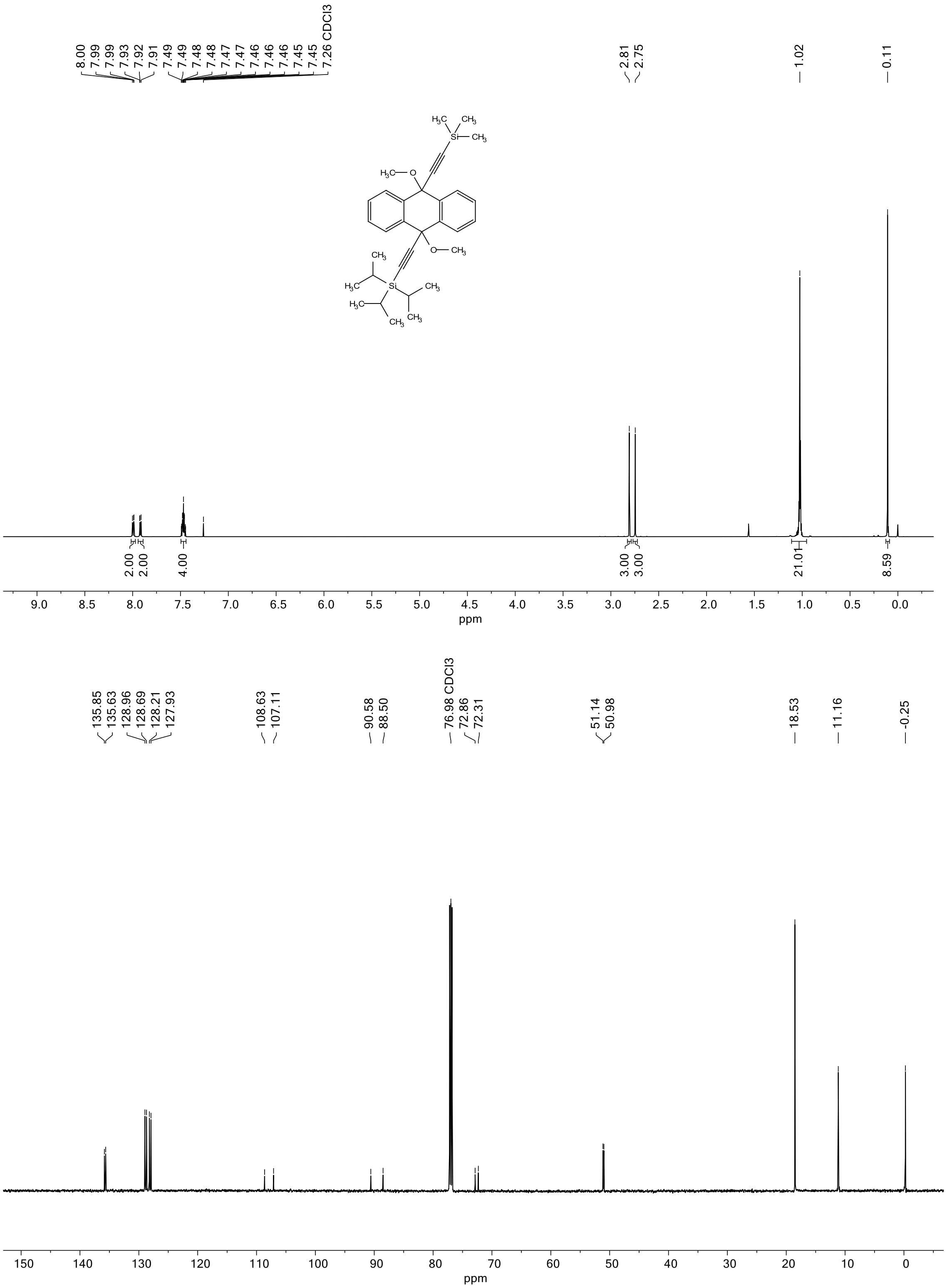

Figure S25. ^{1}H NMR (top) and ^{13}C NMR (bottom) spectra of **S2** in $CDCl_3$.

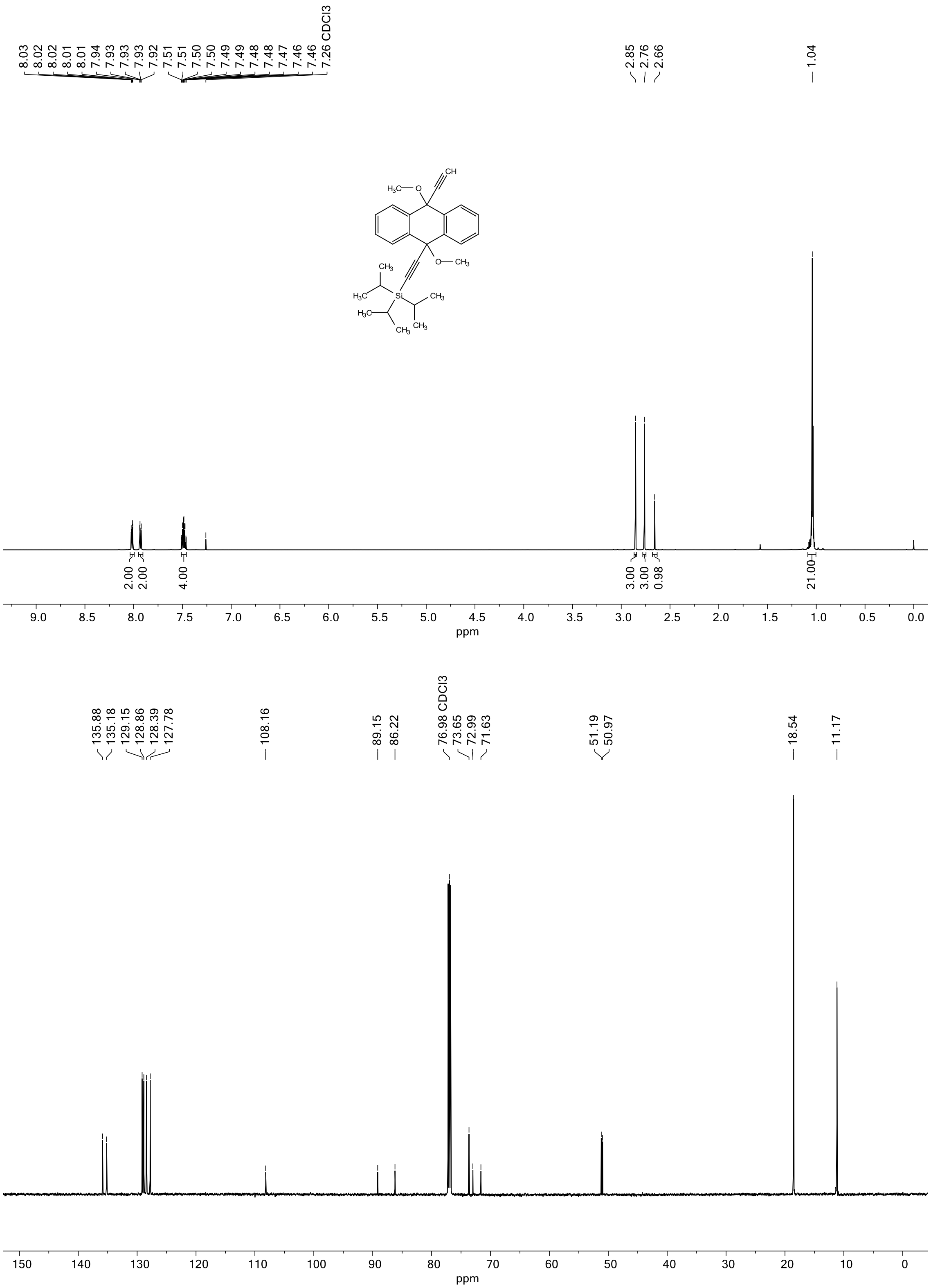

Figure S26. ^{1}H NMR (top) and ^{13}C NMR (bottom) spectra of **S3** in $CDCl_3$.

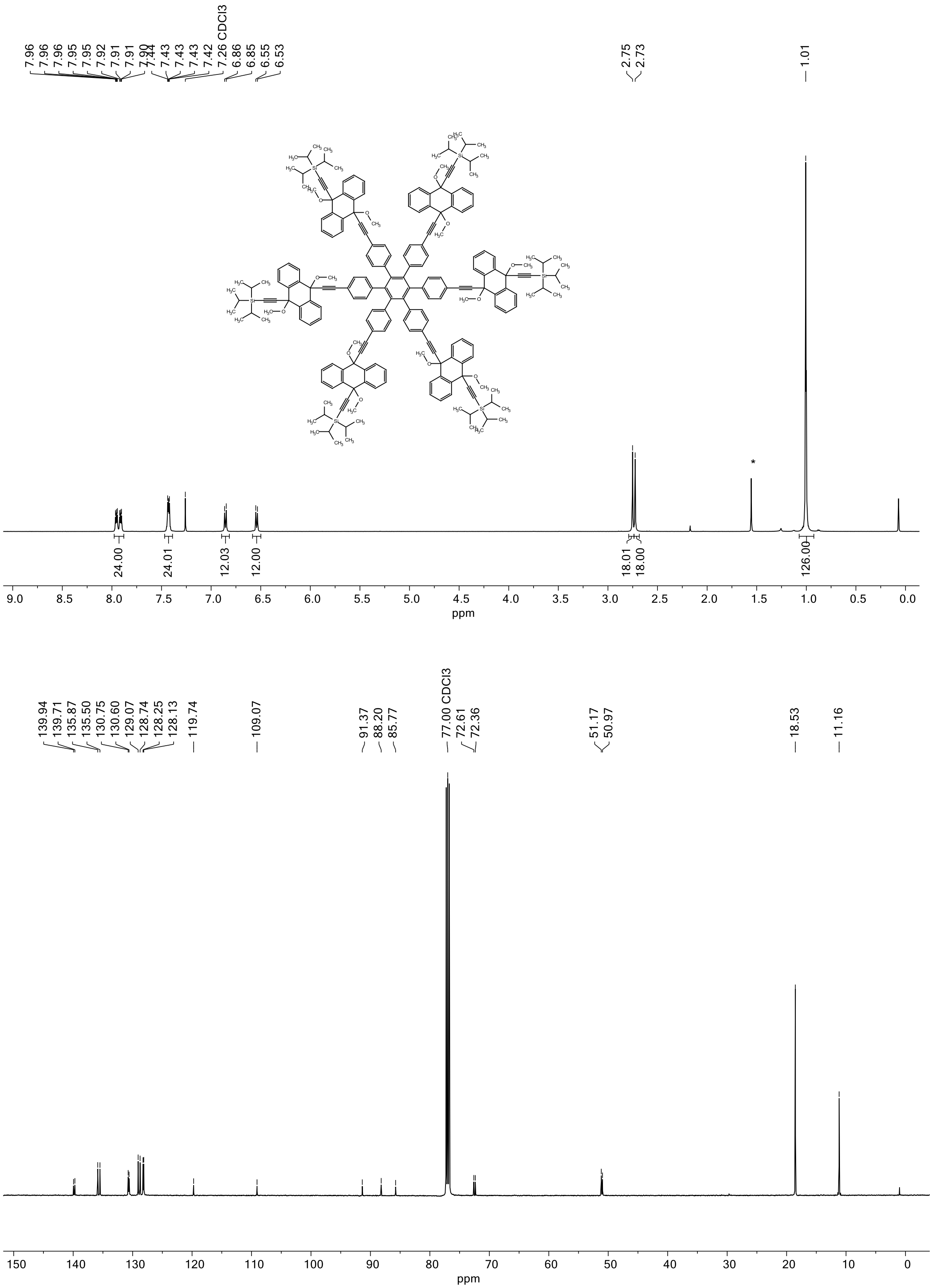

Figure S27. ^{1}H NMR (top) and ^{13}C NMR (bottom) spectra of **S4** in $CDCl_3$.

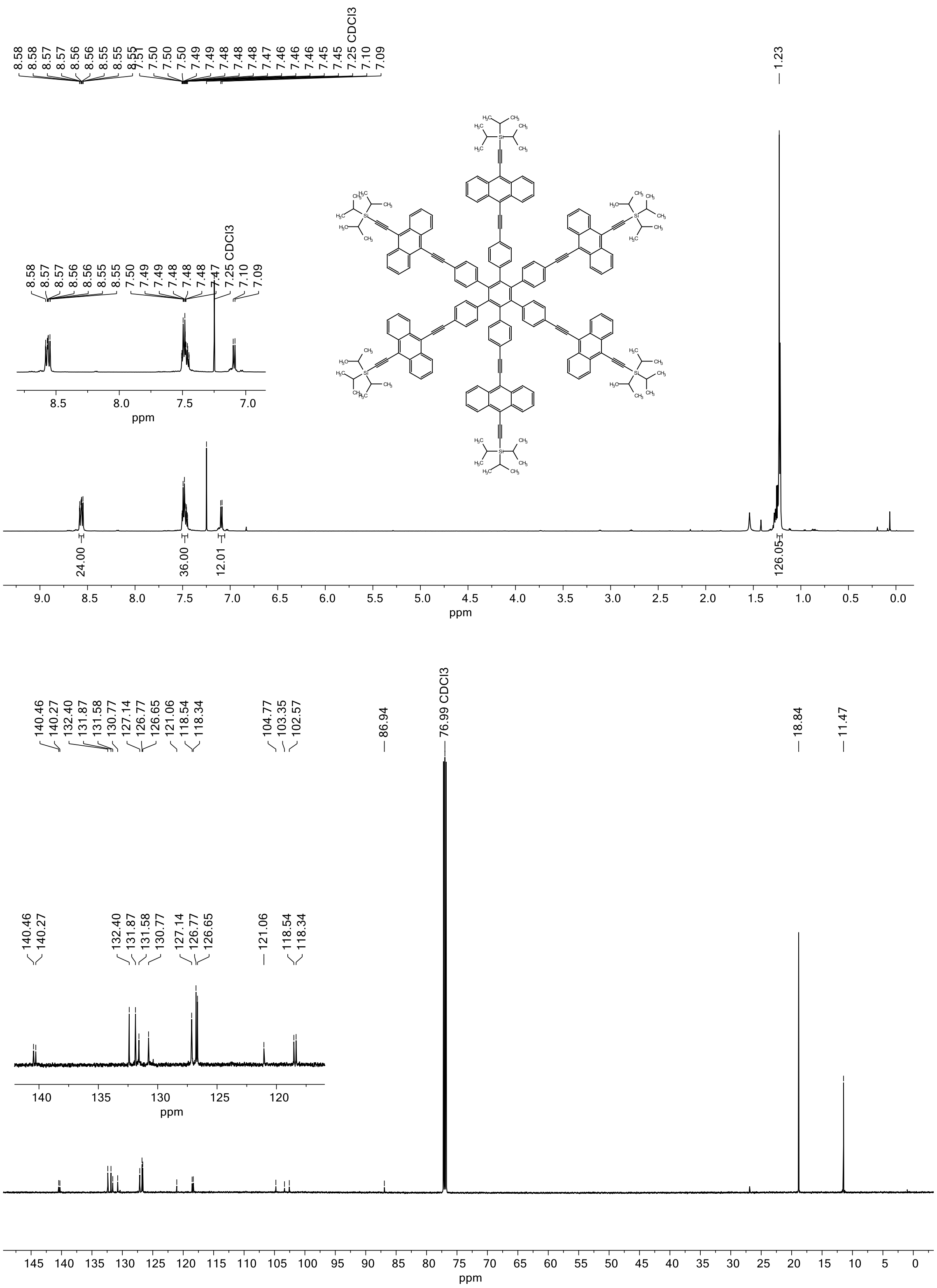

Figure S28. ^{1}H NMR (top) and ^{13}C NMR (bottom) spectra of **S5** in $CDCl_3$.

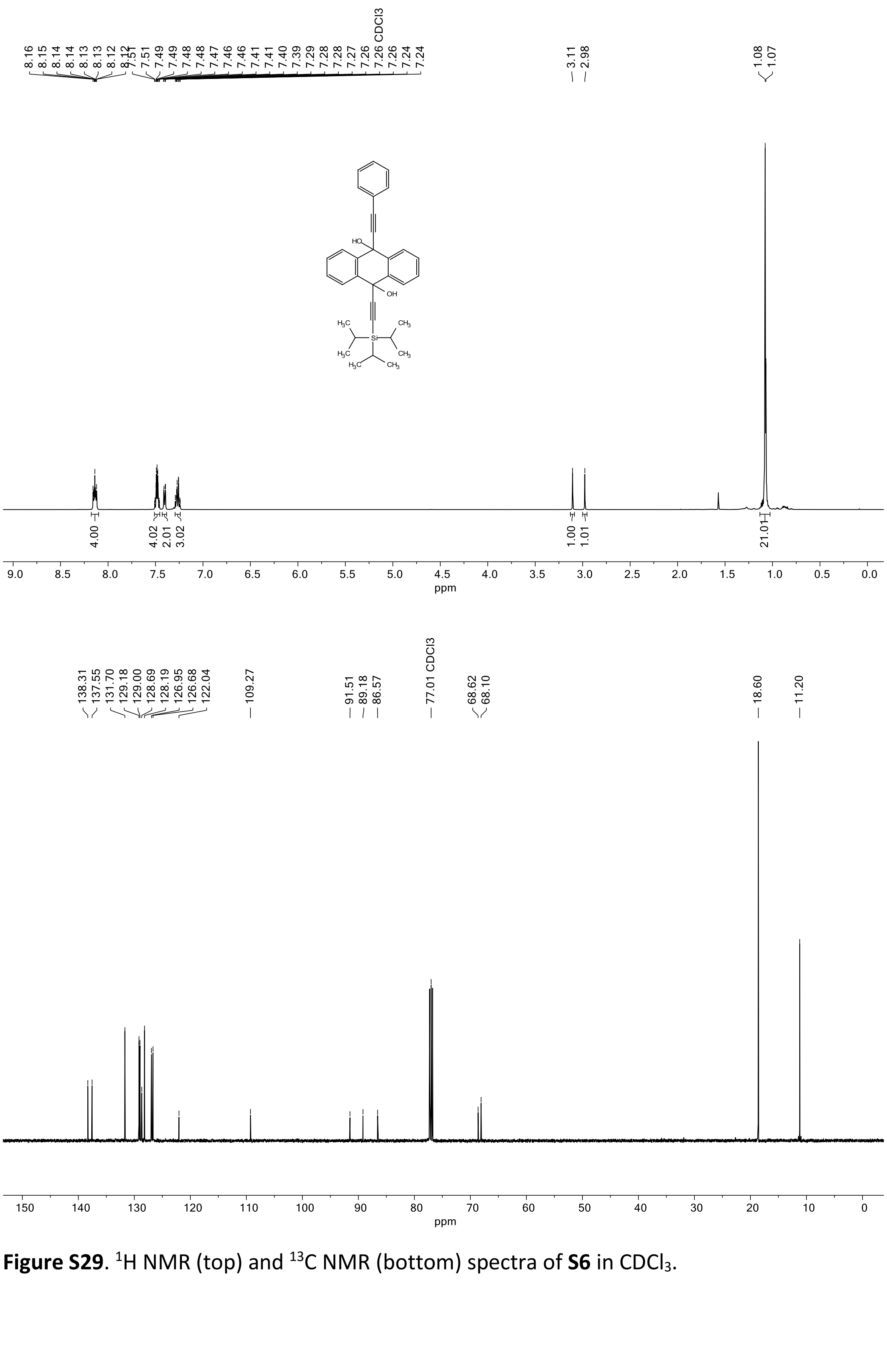

Figure S29. ^{1}H NMR (top) and ^{13}C NMR (bottom) spectra of **S6** in $CDCl_3$.

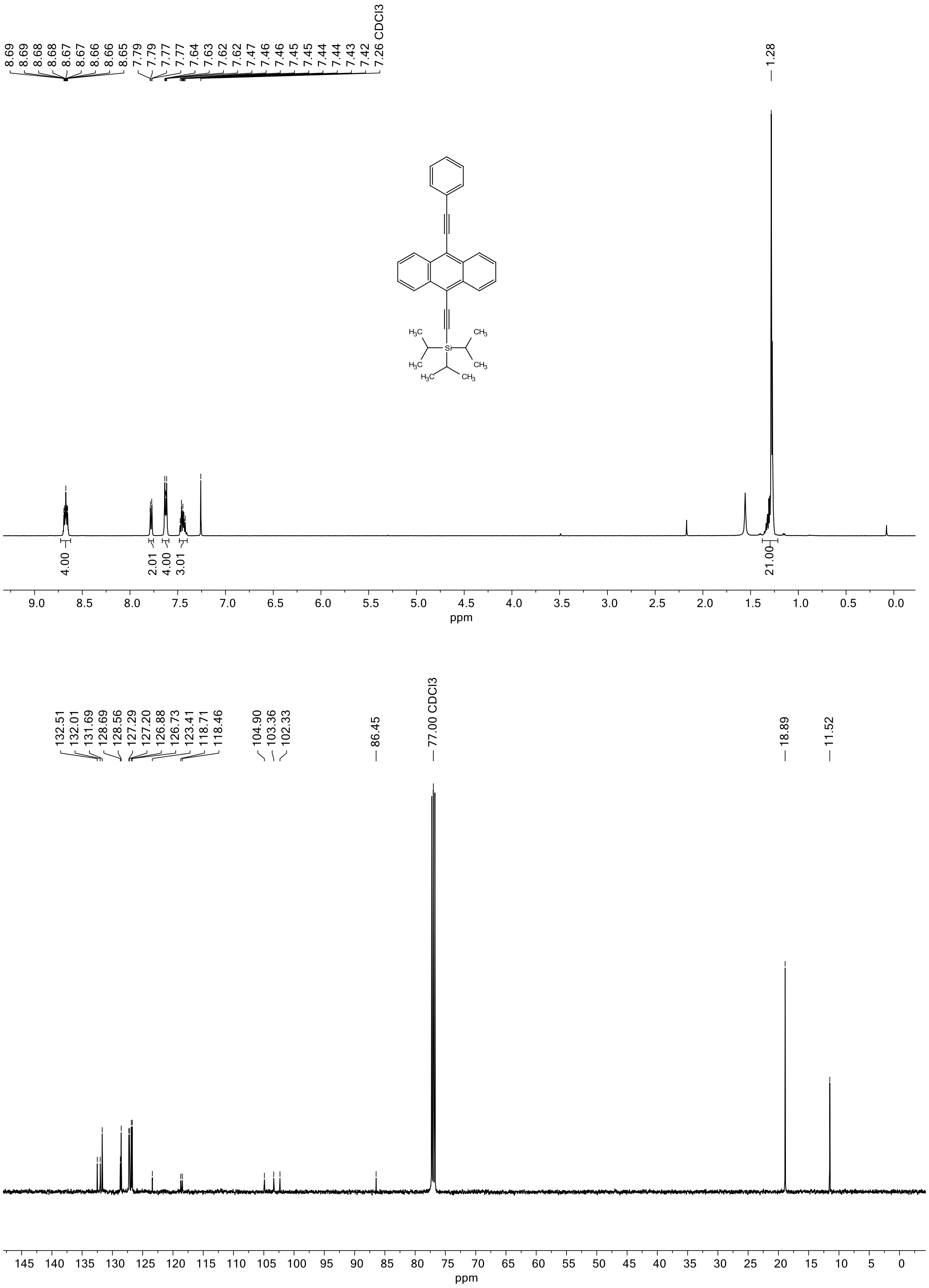

Figure S30. ^{1}H NMR (top) and ^{13}C NMR (bottom) spectra of **S7** in $CDCl_3$.

References:

(1) Radiunas, E.; Raišys, S.; Juršėnas, S.; Jozeliūnaitė, A.; Javorskis, T.; Šinkevičiūtė, U.; Orentas, E.; Kazlauskas, K. Understanding the Limitations of NIR-to-Visible Photon Upconversion in Phthalocyanine-Sensitized Rubrene Systems. *J. Mater. Chem. C* **2020**, *8* (16), 5525–5534. https://doi.org/10.1039/C9TC06031F.

(2) Baronas, P.; Elholm, J. L.; Moth-Poulsen, K. Efficient Degassing and Ppm-Level Oxygen Monitoring Flow Chemistry System. *React. Chem. Eng.* **2023**, *8* (8), 2052–2059. https://doi.org/10.1039/D3RE00109A.

(3) Wu, G.; Cao, E.; Kuhn, S.; Gavriilidis, A. A Novel Approach for Measuring Gas Solubility in Liquids Using a Tube-in-Tube Membrane Contactor. *Chem. Eng. Technol.* **2017**, *40* (12), 2346–2350. https://doi.org/10.1002/ceat.201700196.

(4) Würth, C.; Grabolle, M.; Pauli, J.; Spieles, M.; Resch-Genger, U. Relative and Absolute Determination of Fluorescence Quantum Yields of Transparent Samples. *Nat. Protoc.* **2013**, *8* (8), 1535–1550. https://doi.org/10.1038/nprot.2013.087.

(5) Setiawan, D.; Kazaryan, A.; Martoprawiro, M. A.; Filatov, M. A First Principles Study of Fluorescence Quenching in Rhodamine B Dimers: How Can Quenching Occur in Dimeric Species? *Phys. Chem. Chem. Phys.* **2010**, *12* (37), 11238–11244. https://doi.org/10.1039/C004573J.

(6) Rurack, K.; Spieles, M. Fluorescence Quantum Yields of a Series of Red and Near-Infrared Dyes Emitting at 600−1000 Nm. *Anal. Chem.* **2011**, *83* (4), 1232–1242. https://doi.org/10.1021/ac101329h.

(7) Bansal, A. K.; Holzer, W.; Penzkofer, A.; Tsuboi, T. Absorption and Emission Spectroscopic Characterization of Platinum-Octaethyl-Porphyrin (PtOEP). *Chem. Phys.* **2006**, *330* (1), 118–129. https://doi.org/10.1016/j.chemphys.2006.08.002.

(8) Nishimura, N.; Gray, V.; Allardice, J. R.; Zhang, Z.; Pershin, A.; Beljonne, D.; Rao, A. Photon Upconversion from Near-Infrared to Blue Light with TIPS-Anthracene as an Efficient Triplet–Triplet Annihilator. *ACS Mater. Lett.* **2019**, *1* (6), 660–664. https://doi.org/10.1021/acsmaterialslett.9b00287.

(9) Naimovičius, L.; Radiunas, E.; Dapkevičius, M.; Bharmoria, P.; Moth-Poulsen, K.; Kazlauskas, K. The Statistical Probability Factor in Triplet Mediated Photon Upconversion: A Case Study with Perylene. *J. Mater. Chem. C* **2023**, *11* (42), 14826–14832. https://doi.org/10.1039/D3TC03158F.

(10) Brückner, C.; Engels, B. Benchmarking Singlet and Triplet Excitation Energies of Molecular Semiconductors for Singlet Fission: Tuning the Amount of HF Exchange and Adjusting Local Correlation to Obtain Accurate Functionals for Singlet–Triplet Gaps. *Chem. Phys.* **2017**, *482*, 319–338. https://doi.org/10.1016/j.chemphys.2016.08.023.

(11) Clercq, D. M. de; Collins, M. I.; Sloane, N. P.; Feng, J.; McCamey, D. R.; Tayebjee, M. J. Y.; Nielsen, M. P.; Schmidt, T. W. Singlet Fission in TIPS-Anthracene Thin Films. *Chem. Sci.* **2024**, *15* (17), 6402–6409. https://doi.org/10.1039/D3SC06774B.

(12) Rogers, J. E.; Nguyen, K. A.; Hufnagle, D. C.; McLean, D. G.; Su, W.; Gossett, K. M.; Burke, A. R.; Vinogradov, S. A.; Pachter, R.; Fleitz, P. A. Observation and Interpretation of Annulated Porphyrins: Studies on the Photophysical Properties of Meso-Tetraphenylmetalloporphyrins. *J. Phys. Chem. A* **2003**, *107* (51), 11331–11339. https://doi.org/10.1021/jp0354705.

(13) Kobayashi, K.; Kobayashi, N.; Ikuta, M.; Therrien, B.; Sakamoto, S.; Yamaguchi, K. Syntheses of Hexakis(4-Functionalized-Phenyl)Benzenes and Hexakis[4-(4′-Functionalized-Phenylethynyl)Phenyl]Benzenes Directed to Host Molecules for Guest-Inclusion Networks. *J. Org. Chem.* **2005**, *70* (2), 749–752. https://doi.org/10.1021/jo048521i.
