# Supplementary material for: Automated Research Platform for Development of Triplet–Triplet Annihilation Photon Upconversion Systems
Source: ACS Cent Sci. 2025 Feb 21;11(3):413–21. doi: 10.1021/acscentsci.4c02059 (PMC11950846; doi:10.1021/acscentsci.4c02059)
Supplement: Supplementary file 2 — oc4c02059_si_002.pdf [file oc4c02059_si_002.pdf]

oc-2024-02059b.R1

Name: Peer Review Information for "Automated Research Platform for Development of Triplet-Triplet Annihilation Photon Upconversion Systems"

First Round of Reviewer Comments

Reviewer: 1

Comments to the Author

In this manuscript, Moth-Poulsen and coworkers describe the use of automation to accelerate the discovery and optimization of triplet-triplet annihilation photon upconversion (TTA-UC) systems, which represent an emergent area of photochemical research. The authors rightfully recognize that empirical selection and optimization of TTA-UC remains challenging for the scientific community and that it serves as a major bottleneck for further advancements in the field. Thus, the premise of this work is meritorious. The authors demonstrate the potential of their automated platform on both known and novel TTA-UC systems, showcasing its versatility. The fundamental knowledge gained from the detailed concentration maps developed by the authors will serve as an excellent guide for future optimization by labs with or without the flow setup used for automation. However, I also expect that given the simplicity of the setup developed by the authors that other labs may be inspired to do the same and use it for optimization of a wider range of TTA-UC systems. I therefore find the work to have a high potential for impact in the field. The main critique I have is that the use of automation to create large maps of concentration dependent performance is not particularly innovative (nor is the specific setup demonstrated by the authors). However, I think that this is counterbalanced by the utility of the new knowledge, as noted previously. Moreover, the authors do a good job of clearly describing the complexities of these systems that go beyond simply altering concentration of the two main components (annihilator and sensitizer), including overlap between annihilator emission and sensitizer absorption and associated inner-filter effects, triplet energy matching, and effects of aggregation in solution. This descriptive text showcases the need for this level of detailed analysis in TTA-UC systems. One other critique is that the scope of the study remains relatively small given the high throughput nature of the setup. Additional examples of known TTA-UC systems as well as varying solvent composition could further strengthen the impact of this work. That stated, keeping it more concise (as is) gives a clear story. Overall, I recommend that this work be considered for publication in ACS Central Science pending minor revisions, with additional comments/questions for the authors to consider provided below.

Comments/questions:

1. It was not immediately clear to me how the authors extrapolated some of the color maps to the bottom left corners of the plots. In some cases, such as Figure 3a, the color gradients continue to change beyond where data was collected. Additional details on the color map generation/extrapolation would be helpful. Alternatively, cutting the color maps more closely to the experimental data points and avoiding extrapolation would alleviate the potential issues.

2. Minor suggestion: The authors clearly note that the upconversion quantum yields are out of a maximum of 50% in the captions of Figures 3 and 4. However, it would be helpful to explicitly state this in the "TTA-UC Parameters" section given that the value is inconsistently reported in the literature to be out of 50% or out of 100% maximum, making it extra important to clearly state. For example, the authors could modify the sentence on page 4 lines 10-12 to end with "... one singlet excitation (i.e., 50% maximum quantum yield)."

3. Minor stylistic suggestions: In Figure 4a/b the authors are encouraged to make the colors for Pd and Pt red and blue instead of green and blue to make the traces more colorblind friendly. They may also consider adding a symbol next to each of the traces in (a) and (b) (such as a square and triangle) to avoid any issues of colorblindness in legibility of the traces.

Reviewer: 2

Comments to the Author

The authors propose a method for screening the efficiency of TTA-UC using a flow system. Generally, TTA-UC requires optimization of multiple parameters, and until now, parameter optimization has only been carried out within a limited range based on researchers' empirical rules. This method is a very useful technique for this field, as it examines the impact on the efficiency of TTA-UC by changing parameters over a wider range and exploring areas that have not been investigated until now. It could play an important role in the development of more types of sensitizers and emitters in the future. I support the publication of this paper if the following technical comments are addressed.

1. As shown by Monguzzi et al., the rate of TTA and natural decay are the same at Ith, but strictly speaking, according to the paper by Murakami et al. (Phys. Chem. Chem. Phys., 2021, 23, 18268-18282), 1/2ΦUC_max is not obtained at Ith. It would be better to use a different, more appropriate calculation method or to specify it as a different parameter.

2. It is interesting that you have mapped the emission output, but it would be easier for readers to understand if you showed the external quantum yield.

3. It is difficult to say that the effect of residual oxygen has been fully understood. In general, the TTA-UC emission intensity changes until the residual oxygen is consumed, but what is the time variation in this method? Also, anthracene derivatives can efficiently remove singlet oxygen, but is it possible to screen other emitters in the same way?

Author's Response to Peer Review Comments:

Dear Editor,

Thank you for considering our manuscript, we were pleased to read the reviewers positive comments, and have done our best to answer the minor revisions that they noted.

Please find here, our point by point response.

Best regards

Kasper, on behalf of all authors

PS we are happy to accept the invitation to submit a journal cover, and have uploaded our suggestion alongside the manuscript files.

Reviewer(s)' Comments to Author:

Reviewer: 1

Recommendation: Publish in ACS Central Science after minor revisions noted.

Comments:

In this manuscript, Moth-Poulsen and coworkers describe the use of automation to accelerate the discovery and optimization of triplet-triplet annihilation photon upconversion (TTA-UC) systems, which represent an emergent area of photochemical research. The authors rightfully recognize that empirical selection and optimization of TTA-UC remains challenging for the scientific community and that it serves as a major bottleneck for further advancements in the field. Thus, the premise of this work is meritorious. The authors demonstrate the potential of their automated platform on both known and novel TTA-UC systems, showcasing its versatility. The fundamental knowledge gained from the detailed concentration maps developed by the authors will serve as an excellent guide for future optimization by labs with or without the flow setup used for automation. However, I also expect that given the simplicity of the setup developed by the authors that other labs may be inspired to do the same and use it for optimization of a wider range of TTA-UC systems. I therefore find the work to have a high potential for impact in the field. The main critique I have is that the use of automation to create large maps of concentration dependent performance is not particularly

innovative (nor is the specific setup demonstrated by the authors). However, I think that this is counterbalanced by the utility of the new knowledge, as noted previously. Moreover, the authors do a good job of clearly describing the complexities of these systems that go beyond simply altering concentration of the two main components (annihilator and sensitizer), including overlap between annihilator emission and sensitizer absorption and associated inner-filter effects, triplet energy matching, and effects of aggregation in solution. This descriptive text showcases the need for this level of detailed analysis in TTA-UC systems. One other critique is that the scope of the study remains relatively small given the high throughput nature of the setup. Additional examples of known TTA-UC systems as well as varying solvent composition could further strengthen the impact of this work. That stated, keeping it more concise (as is) gives a clear story. Overall, I recommend that this work be considered for publication in ACS Central Science pending minor revisions, with additional comments/questions for the authors to consider provided below.

We sincerely thank the reviewer for recognizing the significance of our work in advancing the discovery of new phenomena in both established and novel TTA-UC systems. While we acknowledge that flow-based automated methods are not entirely new, specific innovations in our setup—such as in-line degassing and attenuated laser excitation—are, to the best of our knowledge, novel when integrated into a comprehensive characterization system. Developing and optimizing this system required several months of meticulous refinement to achieve optimal performance. Given the substantial time and effort invested in designing and thoroughly testing the automated system, we chose to focus our investigation on two well-known and widely-used in the literature systems in a commonly used solvent, along with two novel systems. This approach clearly demonstrates the system's precision and capability to detect weaker upconverted emissions while providing a robust foundation for future studies on a broader range of systems.

Comments/questions:

1. It was not immediately clear to me how the authors extrapolated some of the color maps to the bottom left corners of the plots. In some cases, such as Figure 3a, the color gradients continue to change beyond where data was collected. Additional details on the color map generation/extrapolation would be helpful. Alternatively, cutting the color maps more closely to the experimental data points and avoiding extrapolation would alleviate the potential issues.

To visualize the 3D data with non-equivalent X and Y coordinates—corresponding to the measured sensitizer and annihilator concentrations—we first interpolated the data onto a fine grid (1000x1000 points). To minimize artifacts introduced during interpolation, we then applied a Gaussian filter (σ=5, order=0) to smooth the data. The resulting smoothed data was used to generate contour plots. The color maps represent interpolated values between the lowest and highest measured points in the XY coordinates. While the extremal points may not appear explicitly in the graphs due to the white background, the whole set of concentration data points are presented in Figure 2b. To enhance clarity, we have supplemented the description of the data plotting procedure in the Supporting Information.

2. Minor suggestion: The authors clearly note that the upconversion quantum yields are out of a maximum of 50% in the captions of Figures 3 and 4. However, it would be helpful to explicitly state this in the "TTA-UC Parameters" section given that the value is inconsistently reported in the literature to be out of 50% or out of 100% maximum, making it extra important to clearly state. For example, the authors could modify the sentence on page 4 lines 10-12 to end with "… one singlet excitation (i.e., 50% maximum quantum yield)."

We thank the reviewer for their suggestion. The recommended phrase has been incorporated into the main text. Additionally, we note that all subsequent upconversion quantum yields were previously verified and, where necessary, adjusted to correspond to a maximum quantum yield of 50%.

3. Minor stylistic suggestions: In Figure 4a/b the authors are encouraged to make the colors for Pd and Pt red and blue instead of green and blue to make the traces more colorblind friendly. They may also consider adding a symbol next to each of the traces in (a) and (b) (such as a square and triangle) to avoid any issues of colorblindness in legibility of the traces.

We sincerely thank the reviewer once again for their valuable suggestion. The colors in Figures 4a and 4b have been updated accordingly.

Reviewer: 2

Recommendation: Publish in ACS Central Science after minor revisions noted.

Comments:

The authors propose a method for screening the efficiency of TTA-UC using a flow system. Generally, TTA-UC requires optimization of multiple parameters, and until now, parameter optimization has only been carried out within a limited range based on researchers' empirical rules. This method is a very useful technique for this field, as it examines the impact on the efficiency of TTA-UC by changing parameters over a wider range and exploring areas that have not been investigated until now. It could play an important role in the development of more types of

sensitizers and emitters in the future. I support the publication of this paper if the following technical comments are addressed.

1. As shown by Monguzzi et al., the rate of TTA and natural decay are the same at Ith, but strictly speaking, according to the paper by Murakami et al. (Phys. Chem. Chem. Phys., 2021, 23, 18268-18282), 1/2ΦUC_max is not obtained at Ith. It would be better to use a different, more appropriate calculation method or to specify it as a different parameter.

We thank the reviewer for this insightful remark. We have defined intensity threshold based on the most recent publication on the subject (Ref 30 in the manuscript). The formal definition of the intensity threshold, as stated by Edhborg et al. in Photochem. Photobiol. Sci. 2022, 21(7), 1143–1158, is:

"...the steady-state excitation intensity at which half of the annihilator triplet population depopulates by TTA. An equivalent definition is the excitation intensity at which the upconversion quantum yield reaches half of its maximum value."

In the same paper, the authors acknowledge:

"...in a recent publication by Murakami et al., it has been shown that the threshold intensity achieved using this method, Ith', does not represent the threshold intensity by the formal definition, Ith. In fact, it is concluded in this publication that the excitation intensity where the lines of slopes 2 and 1 intersect corresponds to half the value of Ith, a conclusion that has been verified by us."

However, acknowledging the ambiguity between two intensity threshold definitions we have replaced Ith with Ith(50%) to represent intensity threshold at half of upconversion quantum yield used in our manuscript.

2. It is interesting that you have mapped the emission output, but it would be easier for readers to understand if you showed the external quantum yield.

The upconversion quantum yields presented in the study actually represent external quantum yields, defined as the ratio of emitted photons to absorbed photons. For more details on the calibration of quantum yields using the relative fluorescence method, please refer to Section 3 in the Supporting Information.

3. It is difficult to say that the effect of residual oxygen has been fully understood. In general, the TTA-UC emission intensity changes until the residual oxygen is consumed, but what is the time variation in this method? Also, anthracene derivatives can efficiently remove singlet oxygen, but is it possible to screen other emitters in the same way?

We acknowledge that anthracene derivatives, such as diphenylanthracene (DPA) used in this work, can in principle act as photochemical oxygen scavengers, as noted in prior studies (Nat. Rev. Chem. 2, 437–452; J. Am. Chem. Soc. 94, 4991–4996). However, in our tests of oxygen scavenging capacity, we observed only a marginal increase (a few percent) in upconverted emission in the PtOEP-DPA solution during the initial minute of irradiation at maximum photon flux, with even

weaker effects at lower photon flux. We believe that more effective photochemical oxygen scavenging agents exist (e.g., Photochem. Photobiol. Sci., 2017, 16, 1327–1334), which could be explored in future iterations of the automated system to enhance its performance further.
